# Blind Predictions of DNA and RNA Tweezers Experiments with Force and Torque

**DOI:** 10.1371/journal.pcbi.1003756

**Published:** 2014-08-07

**Authors:** Fang-Chieh Chou, Jan Lipfert, Rhiju Das

**Affiliations:** 1 Department of Biochemistry, Stanford University, Stanford, California, United States of America; 2 Department of Bionanoscience, Kavli Institute of Nanoscience, Delft University of Technology, Delft, The Netherlands; 3 Department of Physics and Center for Nanoscience (CeNS), University of Munich, Munich, Germany; 4 Biophysics Program, Stanford University, Stanford, California, United States of America; 5 Department of Physics, Stanford University, Stanford, California, United States of America; Chapman University, United States of America

## Abstract

Single-molecule tweezers measurements of double-stranded nucleic acids (dsDNA and dsRNA) provide unprecedented opportunities to dissect how these fundamental molecules respond to forces and torques analogous to those applied by topoisomerases, viral capsids, and other biological partners. However, tweezers data are still most commonly interpreted *post facto* in the framework of simple analytical models. Testing falsifiable predictions of state-of-the-art nucleic acid models would be more illuminating but has not been performed. Here we describe a blind challenge in which numerical predictions of nucleic acid mechanical properties were compared to experimental data obtained recently for dsRNA under applied force and torque. The predictions were enabled by the HelixMC package, first presented in this paper. HelixMC advances crystallography-derived base-pair level models (BPLMs) to simulate kilobase-length dsDNAs and dsRNAs under external forces and torques, including their global linking numbers. These calculations recovered the experimental bending persistence length of dsRNA within the error of the simulations and accurately predicted that dsRNA's “spring-like” conformation would give a two-fold decrease of stretch modulus relative to dsDNA. Further blind predictions of helix torsional properties, however, exposed inaccuracies in current BPLM theory, including three-fold discrepancies in torsional persistence length at the high force limit and the incorrect sign of dsRNA link-extension (twist-stretch) coupling. Beyond these experiments, HelixMC predicted that ‘nucleosome-excluding’ poly(A)/poly(T) is at least two-fold stiffer than random-sequence dsDNA in bending, stretching, and torsional behaviors; Z-DNA to be at least three-fold stiffer than random-sequence dsDNA, with a near-zero link-extension coupling; and non-negligible effects from base pair step correlations. We propose that experimentally testing these predictions should be powerful next steps for understanding the flexibility of dsDNA and dsRNA in sequence contexts and under mechanical stresses relevant to their biology.

## Author Summary

DNA and RNA are fundamental molecules in the central dogma of molecular biology. Many biological behaviors of double-stranded DNA and RNA – including transcription/translation by proteins and packaging into compact structures – depend on their ability to flex and twist. Single-molecule tweezers now provide accurate mechanical measurements of DNA and RNA helices under force and torque but have not been used to rigorously falsify and thereby advance computational models. Here we present the first such blind challenge, involving recent dsRNA tweezers data that were kept hidden from modelers and a new HelixMC toolkit that resolves challenges in simulating long double helices from base-pair level models. The predictions gave excellent agreement with bending and stretching measurements of dsRNA but failed to recover twisting properties, pinpointing a critical area of future investigation. HelixMC also predicted that poly(A)/poly(T) and Z-DNA–biologically important variants whose elastic responses have not been studied with tweezers–will have distinct mechanical properties. These results open a route to iteratively falsifying and refining computational models of long nucleic acid helices, as is necessary for attaining a predictive understanding of their biological behaviors.

## Introduction

Nucleic acids play central roles in biological processes including transcription, translation, catalysis and regulation of gene expression [1], [2]. Double-stranded RNA and DNA (dsRNA and dsDNA) stretch and twist when interacting with proteins [3], [4] and when forming compact structures such as nucleosomes [5] and packaged viruses [6], [7]. Understanding such deformations is critical for a fundamental understanding of nucleic acids in their biological contexts and for efforts to rationally engineer nanostructures built from dsRNA and dsDNA helices. High precision experimental data are becoming increasingly available from measurements using optical and magnetic tweezers [8]–[20] that measure end-to-end lengths and linking numbers of kilobase-length single molecules upon variation of solution condition, sequence, applied force and torque. In principle, these data offer rigorous challenges that can falsify or validate – and thereby advance – models of nucleic acid flexibility. However, such direct comparison of model predictions and experimental observables remains incomplete.

On one hand, fits to analytical equations based on worm-like chain (WLC) or elastic rod models are in common use for interpreting single-molecule manipulation data [14], [21]–[24], but they lack the power of predicting new experimental results and involve numerous approximations (see below). On the other hand, high-resolution approaches that integrate all-atom energy functions and crystallographic knowledge [25]–[32] offer the prospect of predictive calculations, but the computational costs to simulate kilobase-scale helices remain prohibitively large. Coarse-grained models, such as the base-pair level models (BPLMs) pioneered by Olson and colleagues [33] as well as models that use reduced representations for each base (rather than base-pair) [34]–[36], provide mesoscopic “bridges” between simple analytical models and atomic-level simulations. In this work, we focus on BPLMs as they have fewer degrees of freedom than single-base level models, enabling efficient calculations, and their parameterization can be more easily refined by the growing data of crystallographic structures [33], [37]–[47]. It is worth noting that BPLM is only expected to be applicable to duplexes at low-to-medium tension. Structural transitions involving breaking of base-pairs or formation of non-canonical base-pair interactions, typical at very high tension, are better modeled with single-base level models [48]–[52].

Despite continuing advances, BPLM simulation methods have not yet been used to make direct comparisons with single-molecule experiments. BPLM simulations have focused on helices up to hundreds of base-pairs, significantly smaller than the kilobase lengths probed in single-molecule experiments at which helix bending and twisting may play significant roles in the measured properties. In addition, BPLM calculations have been primarily developed for B-DNA duplexes; growing crystallographic knowledge for dsRNA helices has not been integrated into the BPLM framework. Finally, accurate methods for computing and constraining the twist, writhe, and link of discrete, open-ended helices have not been established until recently [53]–[56] and have not been integrated into BPLM modeling.

Here, we describe a blind prediction challenge, where developers of modeling algorithms (FCC, RD) predicted unreleased data on the mechanical properties of dsDNA and dsRNA helices measured by a team of experimenters (Lipfert et al., unpublished data). More specifically, the torsional properties and stretch modulus of dsRNA have not been previously reported (only the bending persistence length of dsRNA was measured previously [13]; the stretch modulus of dsRNA was published during the modeling [20]). This challenge motivated the development of a software package HelixMC, first presented in this work, to close the methodological gaps described above and thus enable simulations of force vs. extension, effective torsional persistence vs. force, link vs. force, and extension vs. link experiments. The goal of calculating actual experimental observables necessitated several systematic studies to check widespread but poorly tested modeling assumptions, including simulation-based validations of the Moroz-Nelson formula for torsional persistence length [21], [22]. Most importantly, the rigorous comparison between blind predictions and data revealed how current BPLMs largely succeed in modeling stretching and bending but apparently miss physics necessary for understanding dsDNA and dsRNA torsional properties. Finally, HelixMC predictions for previously unmeasured properties of two biological important variants, poly (A)/poly (T) dsDNA and Z-DNA, delineate future experiments that will allow incisive evaluation and revision of current modeling approaches.

## Results

### Brief overview of the simulation

Before presenting the results of the blind prediction, we present an overview of the simulation system and algorithm. Detailed descriptions are given in the Methods section. BPLMs [33], [37]–[46] abstract the entire duplex into multiple base-pairs stacking on top of each other. The coordinate transformation between two neighbor base-pairs (i.e. a base-pair step) is conventionally described with six standard step parameters (shift, slide, rise, tilt, roll, and twist). The internal interactions between neighbor base-pairs can therefore be described using the distribution of these parameters drawn from the Protein Data Bank (PDB) in six-dimensional (6D) space. Typically, these 6D distributions are approximated with 6D multivariate Gaussians to allow continuous sampling of the conformation space. We also tested an alternative scheme which samples directly from existing parameters in the database, without assuming Gaussianity.

The duplexes, represented in BPLM, are then simulated with a Metropolis Monte Carlo (MC) method, with stretching forces and torsional constraints incorporated into the energy function. By default we simulated dsDNA/dsRNA of 3,000 base-pairs at room temperature (298K). At the end of each cycle of Monte Carlo updates, the helix extension and the linking number are recorded. For direct comparison to single molecular tweezers analysis, these data from simulations at different forces and torsional constraints are then used to compute global mechanical properties including bending persistence length, stretch modulus, torsional persistence length and link-extension coupling, by fitting to analytical equations based on the elastic rod model.

### Setup of blind prediction challenges

Single-molecule tweezers experiments allow accurate measurements of the extension and the linking number of long molecules under externally applied stretching forces and torques. Typical experiments include force vs. extension, effective torsional persistence vs. force, link vs. force, and extension vs. link measurements. The published literature on dsDNA mechanical measurements is extensive (see e.g. [10], [11], [18], [19]), but magnetic tweezers data directly probing the torsional properties of dsRNA had not been published at the time of this study (only the bending of dsRNA has been previously studied [13]). Instead, a comprehensive experimental portrait (Lipfert et al., unpublished data) had been acquired by one of us with colleagues but was not publicly released. This situation therefore permitted blind prediction tests of the BPLM approach. Our modeling challenges were to simulate the different experimental setups, to test the applicability of phenomenological formulae used for curve-fitting, and to make quantitative predictions with estimated errors for the following standard constants: bending persistence length *A*, stretch modulus *S*, torsional persistence length *C*, and link-extension coupling *g*.

### Accurate recovery of helix bending

**Figure 1:**
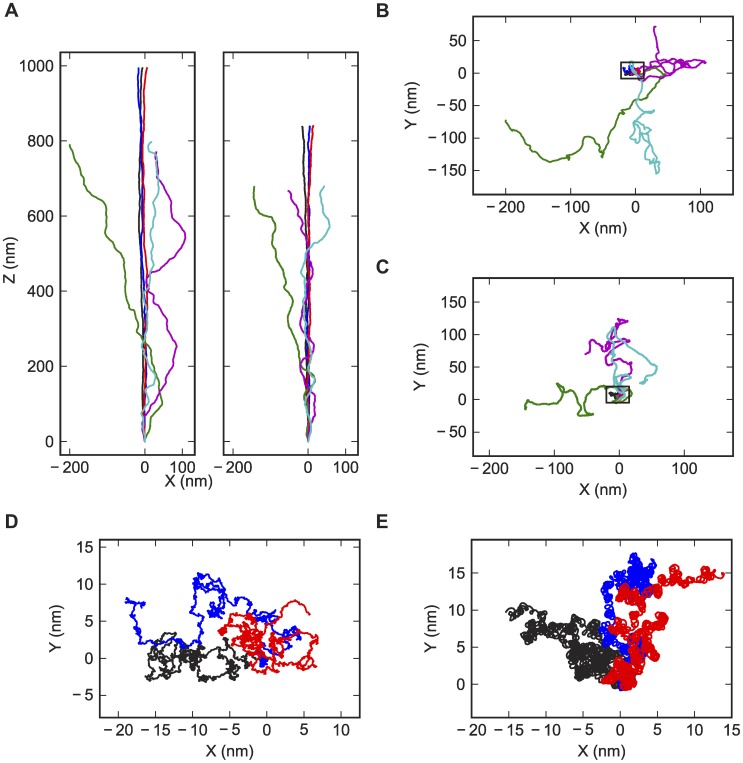
Visualizations of sample conformations from the simulations. Only the axis curve of the helices are shown. Black, red and blue lines: conformations from simulations at 40 pN stretching force. Green, cyan and magenta lines: conformations from simulations at 0.4 pN stretching force. (A) Side view (projection on XZ plane). Left: DNA. Right: dsRNA. (B, D) DNA conformations. (C, E) dsRNA conformations. (B, C) Top view (projection on XY plane). (D, E) Top view showing only the 40 pN simulations.

Drawing on extensive prior work [33], [54], [56], we were able to simulate dsDNA (for validation of the algorithm) and dsRNA (for blind prediction) under applied force using HelixMC. Fig. 1 gives example simulation frames with random sequences, with BPLMs parameterized on crystallographic data with diffraction resolutions better than 2.8 Å and without proteins. (Other BPLM variants are described below.) For both dsDNA and dsRNA, higher stretching force leads to longer end-to-end extensions and smaller fluctuations orthogonal to the stretching direction, qualitatively consistent with theoretical predictions and experimental observations.

**Figure 2:**
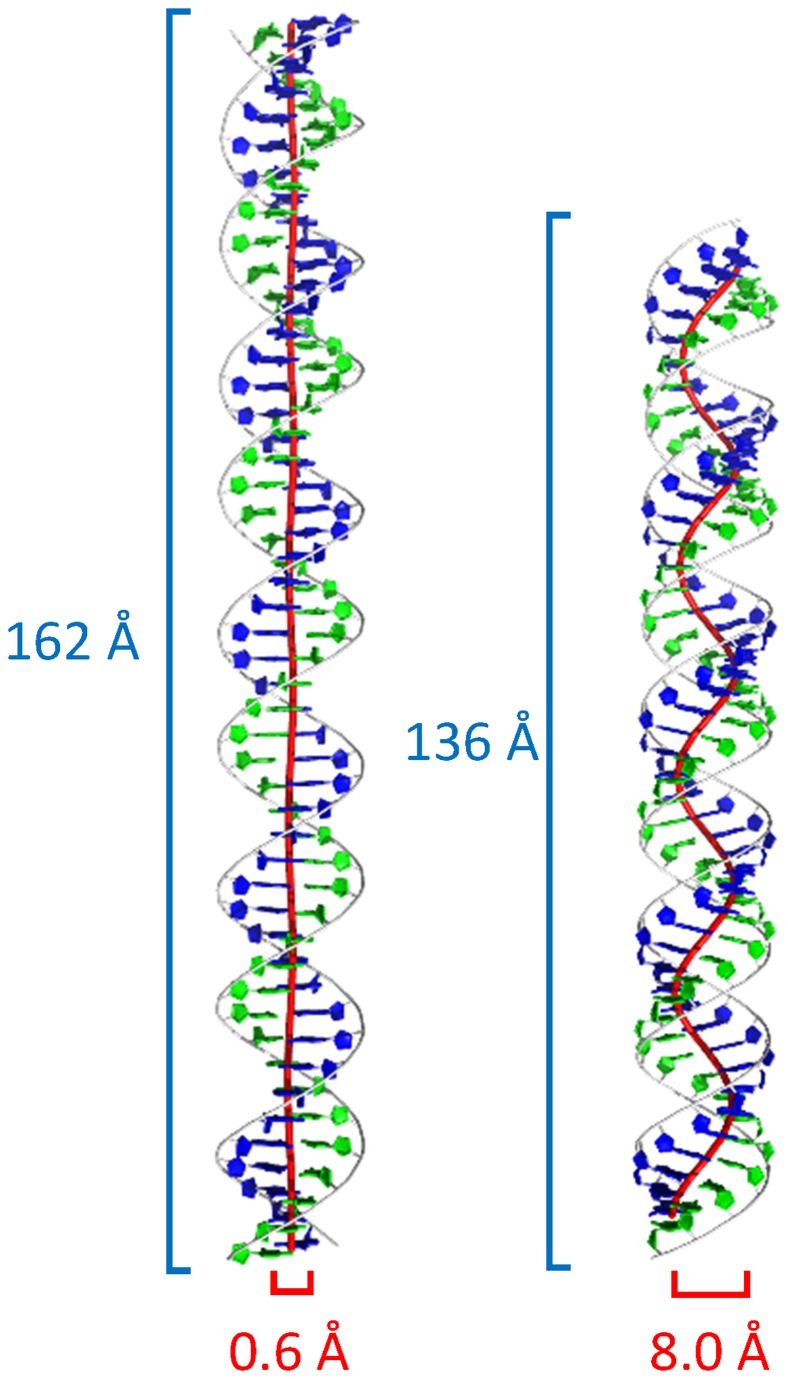
Structures and axis-curves for ideal dsDNA and dsRNA. Left: DNA. Right: dsRNA. The length of the helices is 50 base-pairs. The red lines are the corresponding axis-curves, forming a spiral. Blue numbers are the vertical length of the axis-curve spirals and red numbers are the diameter of the spirals. The axis curve for dsRNA is more “springy” than DNA.

Measurements of the mean end-to-end extension as a function of force give quantitative data for how nucleic acid helices bend, and we first tested if HelixMC recovered the bending persistence length seen in experiments for dsDNA. The simulated data fit well to standard models used in interpreting tweezers experiments, including the extensible worm-like chain (WLC) model proposed by Bouchiat et al. [23] (Fig. 2A; *A* = 54.7±0.6 nm), the inextensible WLC model [23] (*A* = 53±1 nm), and an alternative extensible WLC fitting model developed by Odijk [57] (*A* = 55±1.0 nm); see Table S1 and Fig. S1. The agreement of all three fits to each other and to more direct estimates of *A* by averaging the base-pair step transforming matrix [33] (*A* = 53.0±1.0 nm) confirmed the robustness of *A* as a comparison metric between experimental and simulated data. To bracket systematic error, we further performed simulations using BPLMs with a high-resolution subset of crystallographic data (2.0 Å vs. 2.8 Å diffraction resolution cutoff), without using a Gaussian approximation for the BPLM distributions, and symmetrizing the base-pair step parameters; these variations gave less than 10% changes in *A* (Table 1 and S2). We did however find that inclusion of protein/DNA crystallographic structures, which include more distorted helical conformations, led to reduction of *A* by 30% to 39 nm. Given this level of systematic error, the agreement of the HelixMC calculation and the experimental value for dsDNA (*A* in the range of 44–49 nm at near-physiological salt concentrations [16], [20], [58], [59]) was reasonable.

**Table 1:** Summary and statistics of the curated base-pair step parameter sets.

	DNA			RNA		
	Default	2.8_all	2.0_noprot	default	2.8_all	2.0_noprot
# of data	2964	32261	1456	4503	28397	1404
Resolution cutoff	2.8	2.8	2.0	2.8	2.8	2.0
Contain proteins?	N	Y	N	N	Y	N
k-means clustering?^1^	Y	N	Y	N	N	N
Shift (Å)^2^	0.00(0.57)	0.00(0.64)	0.03(0.59)	0.00(0.57)	0.05(0.66)	−0.02(0.60)
Slide (Å)	0.32(0.86)	−0.12(0.82)	0.35(0.92)	−1.58(0.39)	−1.62(0.44)	−1.58(0.41)
Rise (Å)	3.30(0.23)	3.30(0.25)	3.29(0.21)	3.22(0.20)	3.25(0.24)	3.23(0.16)
Tilt (degree)	−0.05(3.56)	0.03(3.85)	0.08(3.36)	0.02(2.86)	0.13(3.51)	−0.09(2.47)
Roll (degree)	1.60(5.17)	3.03(6.25)	1.76(5.53)	7.89(4.33)	7.71(5.20)	8.18(4.06)
Twist (degree)	35.21(6.24)	33.87(5.42)	35.18(6.44)	31.72(4.25)	31.78(4.73)	31.69(4.36)

1 For removing A-DNA conformation. Only for DNA parameter sets.

2 First value is the mean of that parameter; second value in the parenthesis is the corresponding standard deviation.

The agreement for dsDNA suggested that the prediction of the dsRNA bending would be similarly accurate. The HelixMC prediction for dsRNA was 66 nm, greater than the value for dsDNA, with a systematic error of ∼30%, again based on an alternative BPLM parameterization including protein/RNA crystallographic models (Table 2 and Fig. 2B). Experimental dsRNA tweezers measurements gave values of *A* = 57±2 nm (Lipfert et al., unpublished data) and 59±3 nm [20], greater than the value for dsDNA and in quantitative agreement with the HelixMC value.

**Table 2:** Mechanical properties calculated from all simulations performed.

Simulations	*A*: bending persistence (nm)	*S*: stretch modulus S(pN)	*C*: torsional persistence length(nm)	Slope of link vs. force (rad/pN)	Slope of extension vs. link (nm/turn)	*g*: link-extension coupling 1 (pN·nm)^1^	*g*: link-extension coupling 2 (pN·nm)^2^
**DNA**							
Experiment^3^	45(2)	1000(200)	109(4)	0.06^4^	0.44(0.1)	−90(20)^4^	−70(20)
default^5^	54.7(0.6)	1956.9(102.1)	28.8(0.1)	0.202(0.001)	0.473(0.015)	−131.0(7.5)	−147.4(9.0)
default_frag^6^	54.5(0.6)	1993.4(106.1)	28.7(0.1)	0.206(0.001)	0.461(0.017)	−135.0(7.9)	−146.3(9.5)
2.8_all	39.4(0.5)	1504.8(90.8)	40.5(0.3)	0.227(0.001)	0.743(0.025)	−156.7(10.6)	−177.9(12.3)
2.8_all_frag	39.1(0.4)	1517.8(78.4)	40.4(0.3)	0.229(0.001)	0.757(0.030)	−158.2(9.2)	−182.9(11.9)
2.0_noprot	50.0(0.5)	2146.7(115.2)	27.0(0.1)	0.218(0.002)	0.483(0.025)	−143.7(8.5)	−164.9(12.2)
2.0_noprot_frag	50.0(0.6)	2122.9(146.0)	27.0(0.2)	0.218(0.001)	0.496(0.014)	−142.4(10.8)	−167.5(12.5)
poly(A)/poly(T) default	111.1(0.8)	4373.4(190.8)	97.7(0.4)	0.040(0.000)	0.307(0.017)	−210.6(9.5)	−214.0(15.1)
poly(A)/poly(T) 2.8_all	38.5(0.4)	2403.1(170.3)	38.1(0.1)	0.086(0.004)	0.351(0.014)	−97.2(8.2)	−134.3(11.0)
poly(G)/poly(C) default	62.0(0.6)	1500.3(59.5)	28.9(0.2)	0.260(0.001)	0.540(0.008)	−123.1(5.4)	−129.0(5.5)
poly(G)/poly(C) 2.8_all	51.5(0.6)	1315.1(57.3)	53.7(0.3)	0.243(0.001)	1.037(0.017)	−188.4(9.5)	−217.0(10.1)
Z-DNA	175.4(1.0)	2618.5(59.0)	126.9(0.3)	0.001(0.000)	0.012(0.015)	−4.0(0.7)	−4.9(6.4)
**RNA**							
Experiment^3^	57(2)	350(100)	100(2)	N/A	−0.85(0.04)	N/A	47(14)
default	66.3(0.9)	979.0(40.5)	53.0(0.2)	0.161(0.001)	0.797(0.011)	−116.5(5.2)	−124.2(5.4)
default_frag	66.5(0.8)	983.1(37.9)	52.7(0.2)	0.161(0.001)	0.802(0.016)	−116.2(4.8)	−125.5(5.4)
2.8_all	46.9(0.7)	776.7(36.5)	42.4(0.3)	0.165(0.001)	0.650(0.020)	−76.7(3.8)	−80.3(4.5)
2.8_all_frag	46.9(0.7)	773.1(35.7)	42.8(0.3)	0.164(0.001)	0.648(0.008)	−76.8(3.8)	−79.8(3.8)
2.0_noprot	76.3(1.1)	996.9(42.2)	49.7(0.2)	0.220(0.001)	1.030(0.009)	−146.7(7.0)	−163.5(7.1)
2.0_noprot_frag	75.8(0.9)	1034.0(39.3)	49.9(0.2)	0.219(0.001)	1.045(0.004)	−151.2(6.5)	−171.9(6.6)
poly(A)/poly(U) default	82.0(0.9)	1354.0(51.3)	57.6(0.3)	0.120(0.001)	0.689(0.014)	−133.8(5.6)	−148.4(6.4)
poly(A)/poly(U) 2.8_all	59.1(0.8)	1049.2(48.5)	69.2(0.3)	0.145(0.001)	0.981(0.026)	−146.7(7.5)	−163.9(8.8)
poly(G)/poly(C) default	86.4(0.9)	1197.1(41.7)	62.9(0.4)	0.179(0.001)	1.050(0.018)	−175.6(7.1)	−200.0(7.8)
poly(G)/poly(C) 2.8_all	51.7(0.7)	706.2(29.4)	34.8(0.1)	0.246(0.003)	0.831(0.006)	−82.1(3.8)	−93.4(4.0)

The values in parenthesis are the corresponding fitting errors.

1 Computed from the slope of link vs. force plots.

2 Computed from the slope of extension vs. link plots.

3 Data from Lipfert et al., unpublished data.

4 Data from Gore et al. Nature 2006 (ref. [14]).

5 See Table 1 for detailed description for each parameter set.

6 “_frag” stands for using fragment picking sampling method.

### Stretch modulus and ‘springiness’

In addition to enabling fits of the bending persistence length *A*, force/extension curves give estimates of the stretch modulus *S*, particularly at high force where the helix is pulled straight without bends. For dsDNA simulations with several variations, the HelixMC calculations gave estimates of *S* = 2000 pN. As with the bending behavior, inclusion of protein/DNA structures produced lower stretch modulus values, corresponding to more flexibility (*S* = 1500 pN; Table 2). These calculations overestimated the experimentally measured value for dsDNA of *S* in the range of 900–1400 pN [20], [60], [61], slightly beyond our estimated error.

**Figure 3:**
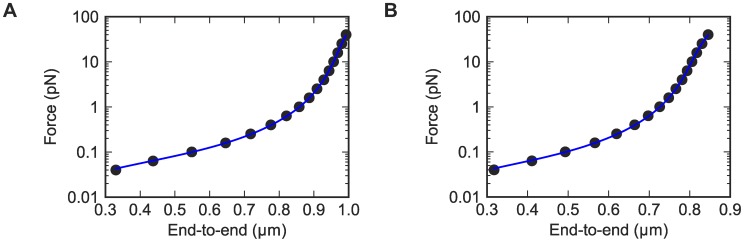
Force vs. extension plots from the simulations. Data are fitted to extensible WLC model by Bouchiat et al. (A) DNA_default. (B) RNA_default.

The HelixMC prediction for the stretch modulus of dsRNA was *S* = 980 pN, with a systematic error of 25%. This estimate was also supported by using an alternative model to fit the simulation stretch modulus (Table S3 and Fig. S1). Given the dsDNA results above, we expected this HelixMC value to overshoot the experimental measurement. Nevertheless, beyond this error in absolute values, we strongly expected that dsRNA would give a relative stretch modulus significantly lower than dsDNA. Unlike the nearly straight axis curve of dsDNA, the base-pair centers of dsRNA trace a ‘spring-like’ axis curve, twirling in circles of radius 8 Å. We developed a novel “springiness” hypothesis, that this “spring-like” property of dsRNA would render it more pliable to stretching, analogous to a spring's lower stretch modulus compared to a straight wire (Fig. 3). Indeed, the experimental measurements for the dsRNA stretch modulus was 350±100 pN (Lipfert et al., unpublished data), more than two-fold less than for dsDNA, in agreement with our prediction. An independent experimental dsRNA measurement released at the time of modeling gave a similar value lower than dsDNA (500–683 pN) [20]. Additional simulation-based tests of the ‘springiness’ hypothesis are described in Supplementary Results and Table S4, S5.

### Discrepancies in torsional persistence length

The development of magnetic tweezers with increasingly sophisticated geometries has enabled torsion-sensitive measurements of dsDNA [16], [62]–[64] and, most recently, measurements on dsRNA that were included in our blind challenge. Before describing the blind comparison, we present HelixMC simulations that were necessary to shed light on puzzling prior results on dsDNA torsional stiffness. Measurements based on topoisomer distributions of closed dsDNA circles, fluorescence polarization anisotropy of intercalated dyes, and x-ray scattering of tethered gold nanoparticles give lower values for torsional persistence length (*C* = 25–80 nm [47], [65]–[68]) than measurements from optical and magnetic tweezers experiments (*C* = 100–120 nm [12], [16], [17], [21], [59]) from several different laboratories and with different tweezers geometries. One potential resolution to these discrepancies is that the apparent torsional stiffness of dsDNA is enhanced beyond its intrinsic value due to tethering constraints that attenuate torsional fluctuations in single-molecule experiments [44]. However, testing this hypothesis has been complicated by a prior inability to integrate link (number of helix turns) in base-pair-level simulations. Additional concerns have stemmed from the poor quality of fits to infer *C* from single molecule experiments with the analytical Moroz-Nelson formula [21], [22], which assumes the Fuller writhe expression and negligible self-avoidance effects.

**Figure 4:**
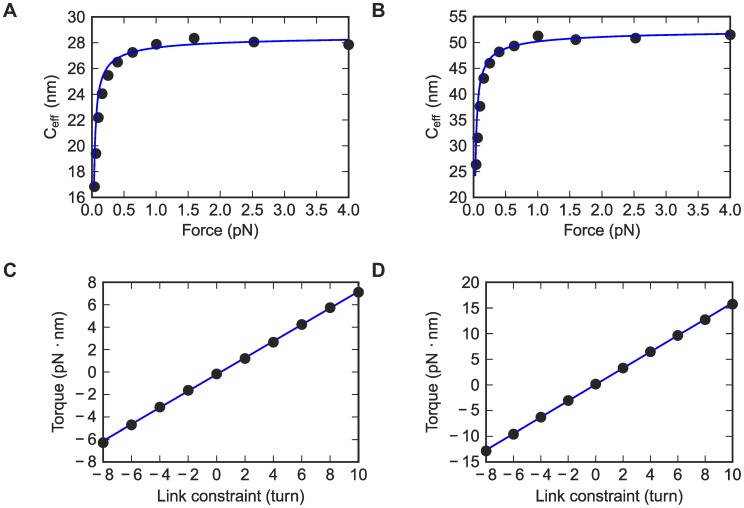
Torsional persistence length from the simulations. (A, C) DNA_default. (B, D) RNA_default. (A, B) Effective torsional persistence lengths at different stretching forces, fitted to Moroz-Nelson model. Only the last six points are used in the fit since Moroz-Nelson model is a high-force expansion. (C, D) Torque vs. the target link constraint plots in link-constrained simulations, at 7 pN stretching forces. Data are fitted to straight lines.

To address these problems, we reasoned that the direct simulations enabled by HelixMC would reveal any systematic overestimation of intrinsic torsional persistence length due to tethering constraints or to the inaccuracy of the Moroz-Nelson model. First, we simulated link fluctuations in dsDNA helices as a function of force, analogous to experiments in references [12], [17], and computed the effective torsional persistence length *C_eff_* by dividing the contour length of the polymer by the variance of the link (Table 2 and Fig. 4A–B). We first observed that the asymptotic value of *C_eff_* (29–40 nm) in our simulation was within error of the ‘intrinsic’ value computed from a normal mode analysis (37.5 nm [43]), suggesting that *C* is not overestimated due to the tethering setup in single molecule experiments. We also tested the effects of *x-y* constraints (perpendicular to the direction of pulling) that might dampen torsional fluctuations, although such constraints are negligible in magnetic tweezers setups (and would also be expected to have a suppressive effect on bending fluctuations). Applying a harmonic *x-y* restoring force with strength of 0.025 pN/nm gave no significant change in *C_eff_* (Fig. S2), disfavoring tether constraints as an explanation for the high *C* anomaly. Second, to test the use of the Moroz-Nelson formula, we fit these simulation data to the Moroz-Nelson model, and found excellent agreement with the same *C* values as described above. The rarity of self-clashing conformations (Supplementary Results and Table S6) and validity of the Fuller writhe formula above 0.4 pN further supported the use of this analytical fit. As a final crosscheck, we also computed the torsional persistence length using the slope of torque vs. number of turns in independent link-constrained simulations at 7 pN, analogous to an alternative experimental approach [16], [59], [62] (Fig. 4C–D, Supplementary Methods). This second simulation method gave torsional persistence length values that agreed well with the first method (within 1%, Table S7), confirming the robustness of the simulation method and Moroz-Nelson fits for inferring *C* in a way that matches experimental procedures.

Given the checks above, the discrepancy between the simulated dsDNA torsional persistence length *C* = 28.8 nm and the value in single molecule experiments *C* = 109 nm cannot be easily explained by systematic errors in the modeling. Furthermore, the deviation of experimental measurements from the Moroz-Nelson formula [16], [17] does not appear to be due to inaccuracies in this phenomenological model, given the successful fits of the model to simulated data. The discrepancies in *C* value and fitting curve strongly indicate either missing physics in modeling dsDNA in both the BPLM and simpler elastic-rod frameworks or currently unknown systematic errors in the experiment (see below, Discussion). Given these issues, we expected that our blind prediction for the torsional persistence length of RNA (*C* = 53 nm) might be an underestimate of the value measured from magnetic tweezers experiment. Indeed the experimental value was two-fold higher, with *C* = 100 nm. However, as with the dsDNA measurements, the Moroz-Nelson formula fit these experimental measurements relatively poorly (Lipfert et al. unpublished data), suggesting that some basic assumption of the BPLM approach is violated (see Discussion below).

### A stringent test from link-extension (“twist-stretch”) coupling

The first measurements of helix mean end-to-end distance versus mean linking number for dsDNA highlighted gaps in theories of DNA elasticity [14], [15]. We thus expected that our final blind challenge, to predict analogous experiments for dsRNA, would provide a highly stringent test for HelixMC and the BPLM approach.

**Figure 5:**
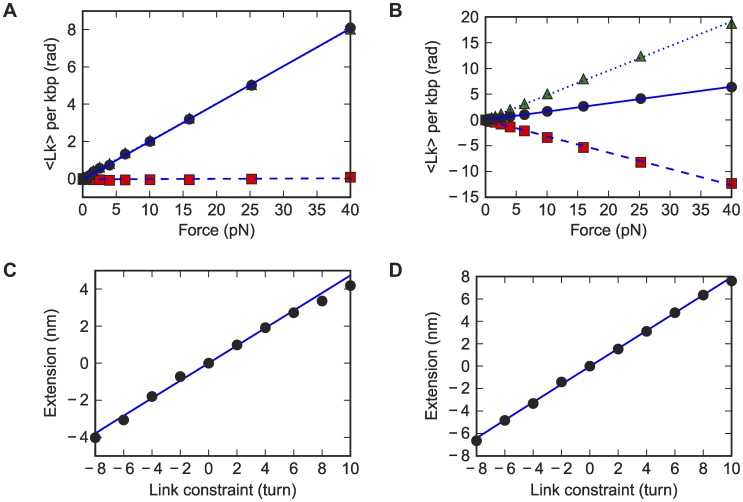
Link-extension coupling constants from the simulations. (A, C) DNA_default. (B, D) RNA_default. (A, B) Link per kbp vs. force plot (black dots) with a linear fit (blue solid lines). The link values are offset such that the first point (at 0.04 pN stretching force) has a link of zero. The corresponding twist (green triangles) and writhe (red squares) component for each link data point, as well as linear fits (dotted and dashed lines) are also shown in the figures. In panel A, the writhe is close to zero, and the link and twist are almost undistinguishable. (C, D) Extension vs. target link constraint in link-constrained simulations with linear fits, at 7 pN stretching forces. The first and last data points are not used for fitting as the linear relationship breaks down at high numbers of turns.

Before presenting the blind comparison, we describe simulation-based tests of assumptions made in the experimental inference of the link-extension coupling *g* (also described as twist-stretch coupling). In previous work, the coupling has been estimated from two different kinds of experiments: (1) stretching the polymer at different forces and observing how the linking number changes in the process [14], [69], and (2) setting up a constant stretching force and observing the polymer's extension as increasing numbers of turns are introduced [14], [15]. In both cases, bending fluctuations at low force (<15 pN) should, in principle, cause deviations from the linear relationships assumed to fit the experimental data (Supplementary Results, Fig. S3, S4). Nevertheless, linear relationships have been empirically observed for link and force (in experiment type 1) and of link and extension (in experiment type 2, but not in experiment type 1) for experiments on dsDNA. Furthermore, linear fits from these independent types of experiments gave consistent results (*g* = −90±20 pN·nm and −70±20 pN·nm, respectively); due to the convention in use, the negative sign corresponds to over-winding of the double helix upon extension (Table 2 and Fig. 5). This empirical relation was indeed confirmed in our simulations. We discovered linear correspondences between dsDNA link and extension in both types of simulated experiments, despite non-linear relationships of the underlying variables. The simulated dsDNA data gave couplings of *g* = −130 pN·nm and −150 pN·nm, respectively, for the two types of experiments, with systematic errors of ±30 pN·nm, based on alternative BPLM parameterizations (Table 2). The dsDNA calculations were therefore in agreement with experimental values within the estimated errors.

For dsRNA, the HelixMC-predicted *g* value was −120 pN·nm (from simulations of both types of experiments), with errors of ±40 pN·nm based on alternative BPLM parameterizations (Table 2 and Fig. 5). This predicted dsRNA value is the same, within error, as the dsDNA simulations. Nevertheless, separation of the link into twist and writhe components in the simulation suggested a different physical picture of link-extension coupling to dsRNA than for dsDNA. The simulated writhe vs. force slope is negative for dsRNA but nearly zero for dsDNA. This effect can be again attributed to the “springiness” of dsRNA axis curve, which carries an intrinsic writhe. Stretching dsRNA unwinds this writhe, while stretching dsDNA has little impact on its already straight axis curve. This behavior would result in a positive link-extension coupling *g* value, opposite in sign to dsDNA. However in the HelixMC dsRNA simulations, the helix twist, the other component of link, rises with extension and overpowers the writhe decrease to produce a net negative link-extension slope, matching the sign of dsDNA simulations.

The dsRNA tweezers experiments gave a value of *g* = +47±14 pN·nm, different from the value given by blind prediction (−120 pN·nm). This discrepancy is well beyond the error associated with different BPLM parameterizations, providing strong evidence against the current BPLM framework for modeling the torsional flexibility of dsRNA. Since the link-extension slope for RNA is a result of cancellation between a positive twist-extension correlation and a negative writhe-extension correlation, the predicted slope is quite sensitive to changes of many of the parameters of the underlying Gaussian potential (Supplementary Results, Table S8, S9). Indeed, by modification of the parameters, we were able to recapitulate the experimentally measured link-extension coupling, as discussed extensively in the experimental paper associated with this work (Lipfert et al., unpublished data). However we note here that this reparameterization is not unique, because the number of parameters (15, for a 6D covariance matrix) is far greater than the number of experimental measurements (four, i.e. bending persistence, stretch modulus, torsional persistence and link-extension coupling).

### Design of future tests

To understand the sequence-dependence of the mechanical properties being studied, and to propose future tests of the BPLM approach, we performed additional simulations of poly(A)/poly(T) and poly(G)/poly(C) for both DNA and RNA (which has U instead of T). Stretches of these homopolymer sequences play critical roles in accessibility of chromatin to RNA polymerase and transcription factors [70], [71]. We also performed simulations on Z-form DNA, which has been hypothesized to occur during DNA transcription to absorb torsional stress [72]. The results are listed in Table 2. For sequence-dependent simulations, we found that for poly(A)/poly(T) DNA, using the default dataset, all the measured mechanical properties increased by 1.5- to 3- fold compared to the random-sequence simulations. However if we used BPLM parameters from the 2.8_all dataset, which includes protein-binding DNA structures, the poly(A)/poly(T) results were not significantly different from the random-sequence results. The difference of predicted stiffness can be explained by the different underlying base-pair step parameters (Supplementary Results, Table S10). We also found smaller but measurable differences between other sequence-specified and random-sequence simulations, and between sequence-specified simulations performed with different base-pair step parameter sets. Further experimental comparisons between sequence-specific and random-sequence DNA/RNA will provide stringent tests of these predictions and to help discriminate which dataset (if any) is more accurate in modeling the sequence-dependence of the mechanical properties.

Simulations of Z-DNA gave dramatically higher bending and torsional persistence lengths (175 nm and 125 nm, respectively) compared to random B-DNA (55 nm and 29 nm, respectively). Again, this higher stiffness is encoded in the underlying step parameters (Supplementary Results, Table S10). Furthermore, the link-extension coupling is estimated to be near zero; this value arises from a complicated cancellation of twist and writhe, and is difficult to explain with simple arguments. Our simulation results agree with data obtained by Thomas and Bloomfield [73] indicating Z-DNA to be much stiffer than B-DNA, with a bending persistence length of 200 nm. However, previous studies on Z-DNA using light scattering, electron microscopy and fluorescence anisotropy have led to inconsistent results, with bending persistence length ranging from 21 to 200 nm and an extremely low torsional persistence length of 7 nm [73]–[75]. These studies did not agree on whether Z-DNA is stiffer then B-DNA. Additional single-molecule tweezers experiments on Z-DNA appear necessary to resolve these issues, and would provide stringent tests of the BPLM approach.

## Discussion

We have presented a set of fundamental tests of how well base-pair level models predict the flexibility of double-stranded nucleic acids, motivated by a desire for improved rigor in this field and by recent single-molecule measurements of dsRNA helices that were blinded to the modelers. A new software package HelixMC that integrates rigorous treatment of twist, writhe, and link allowed direct simulations of dsDNA and dsRNA tweezers experiments with base-pair level models. By fitting the simulated observables with the same analytical models used in experimental measurements, we were able to make direct comparisons of simulation and theory for properties including the bending persistence length, stretch modulus, torsional persistence length and link-extension coupling. We obtained predictions that match some experimental observations, particularly in the ratios of dsRNA to dsDNA values for mechanical properties like bending persistence length. However, we observed quantitative discrepancies for torsional persistence length at high force and the incorrect sign of the link-extension coupling constant for dsRNA. An extensive set of simulations checked that assumptions such as the effects of tethering, the Moroz-Nelson model of torsional persistence length, the curation of the database used to parameterize the BPLM, and the fitted relation of force and link could not account for these discrepancies.

The discrepancies between the BPLM model and tweezers measurements could be due to at least five reasons. First, electrostatic repulsion may account for some discrepancies, but it is difficult to see how corrections needed to increase the torsional stiffness of simulations by three-fold would not also substantially increase the simulated bending stiffness beyond the current values, which agree well with experiments. Experiments with different ionic conditions (particularly highly screening conditions) would help bound these effects. A second possibility is that the base-pair step distributions observed in crystallized nucleic acids do not reflect the fluctuations of nucleic acids in solution [47]. In this case, however, neither a simple overall scaling nor the parsimonious adjustment of a few parameters suffices to bring simulated data into agreement with experiments. Large changes in multiple BPLM parameters are required, in different directions for dsDNA vs. dsRNA and beyond the systematic deviations seen in different curated crystallographic databases, especially to account for a sign change in dsRNA link-extension coupling while retaining the experimental value for dsDNA link-extension coupling (Supplementary Results and Table S8, S9). A third explanation might involve thermal fluctuations involving bulges or non-Watson-Crick pairs, as have been resolved recently albeit with rare population [76]; the population of these alternative structures could be potentially enhanced during torsional stress. Due to the energetic cost of such fluctuations, we would predict that they would lead to a strong temperature dependence of torsional properties. Fourth, the conformation of each base-pair step may affect neighboring base-pair steps. Recent Au-SAXS scattering experiments and crystallographic analyses have suggested the importance of such correlations [47], [77]. Preliminary tests with multi-base-pair fragments in HelixMC indicate that such correlations may have up to 2-fold effects on predicted tweezers-measured properties (Supplementary Results and Fig. S5, S6).

A final explanation for the discrepancy involves the applied tension in single molecule tweezers experiments. On one hand, the tweezers data at low force (<5 pN) are used to infer the bending persistence length *A* and low-force effective torsional persistence lengths *C_eff_*. These parameters are sensitive to both bending as well as intrinsic torsional persistence length via fluctuations captured by the Moroz-Nelson model. In this low force regime, BPLM gives predictions for both parameters with less-than-two-fold discrepancies, for both dsDNA and dsRNA. On the other hand, forces higher than 4 pN are required to suppress bending fluctuations and thereby to isolate stretch modulus *S*, intrinsic torsion persistence length *C*, and link-extension coupling *g*. For these values, the BPLM predictions do not agree with dsDNA or dsRNA measurements. Indeed, there is a more fundamental discrepancy: while the Moroz-Nelson model accounts for the predicted torsional persistence length vs. force from BPLM calculations over a wide range of model parameters, the experimental measurements of *C_eff_* at forces >2 pN cannot be fit by this analytical model. These high-force discrepancies could be rationalized by a model in which tensions greater than 1 pN favor structural states that are more pliant to stretching but torsionally stiffer than the ensemble of conformations seen in crystallized dsRNA and dsDNA. Nucleic acids in solution under constant tension or strong torque, as might be provided by solution-based tweezers [78] or circularization, may enable bulk experimental methods like NMR or Au-SAXS to test this model. It is also possible that single-molecule tweezers experiments on alternative polymers such as poly(A)/poly(T) or Z-form DNA (simulated above) will agree well at all forces with BPLM predictions and thereby offer a baseline for comparison to the mixed sequence dsDNA and dsRNA cases. Alternatively if atomic-level computational methods could predict the structure of the putative weakly stretched state and design sequences or atomic modifications that favor it, the HelixMC toolkit should be able to integrate predictions for long helices that can then be precisely tested through future tweezers experiments.

## Methods

### System setup and summary of the algorithms

The BPLM framework has been described in detail in previous studies [33]. Briefly, each base pair in the nucleic acid is represented by a vector representing the base-pair center and by a coordinate frame representing the orientation of the base-pair [42]. The degrees of freedom of the system are the base-pair steps, defined by the transformation of coordinates from one base-pair to the next base-pair. Each step is described by six parameters (shift, slide, rise, tilt, roll and twist) [79]. The transformation of the step parameters to Cartesian coordinates follows the Calladine and El Hassan Scheme (the CEHS definition) [80], which is also the convention used in the 3DNA package [81], [82]. The ‘technical details’ section of the 3DNA manual offers comprehensive examples of this scheme.

In HelixMC, the origin and the frame of the first base-pair is placed at the origin of the global coordinate system. That is, the base-pair center is placed at the coordinate origin; the normal vector of the base-pair is aligned with the *z*-axis; and the long-axis of the base-pair lies on *y*-axis. In terms of experimental setup, this placement is analogous to fixing one end of the nucleic acid to a surface (i.e. the *xy*-plane in our simulation), an approach routinely employed in magnetic and optical tweezers studies.

Once the origin and the frame of the first base-pair are set, the coordinates of the entire helix can be computed from the six base-pair step parameters. In HelixMC, the conformation of helix is stored and updated in this space of the step parameters, instead of in the Cartesian space. This is similar to describing protein conformations with the internal torsion angles instead of using the Cartesian coordinates of the atoms. For each base-pair step, we assumed the six step parameters form a multivariate normal distribution, of which the parameters were derived by surveying the existing RNA crystal structures (see below). This assumption is equivalent to assuming that positions and orientations of adjacent base-pairs are constrained by a six-dimensional harmonic potential [33].

In this work, the BPLM system was simulated using the Monte Carlo (MC) algorithm. A typical MC run consists of tens of thousands of cycles. A sample, which includes the current extension and linking number of the helix, was extracted at the end of each cycle (i.e. number of cycles equals to number of samples in the simulation). For each cycle, the base-pair steps of the entire helix was updated sequentially starting from the first base-pair step. For each update, a proposed move was generated by modifying only the conformation of the target base-pair step, while keeping the conformation of the rest of the helix intact. Note that the term “conformation” here refers to the six step parameters of each base-pair step, not the Cartesian coordinates of the base-pairs. Because we assumed the step parameters follow a multivariate normal distribution, this proposed conformational move can be efficiently achieved by drawing a random sample from the distribution.

The standard Metropolis criterion [83] was then used to whether to accept the proposed MC move:
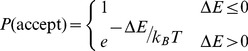
(1)Here *ΔE* equals the energy after the proposed move minus the energy of the initial conformation, *T* is the temperature and *k_B_* is the Boltzmann constant. Because the internal interactions between the base-pair steps are included in the multivariate Gaussian sampling, the *ΔE* in Eq. (1) only reflects the applied torque and force, as described next. For cases where external forces and torques are absent (free helix), the *ΔE* is always zero and the acceptance rate is 100%. For cases with external forces and torques, since each update is applied to one base-pair step only, the new proposed conformation is usually similar to the previous conformation. Therefore the acceptance rates are reasonable in the force and torque range used in this work (8% (40 pN) to 55% (1 pN) for dsDNA, Table S11).

We performed two types of simulations. In the first type of simulation, a stretching force along the *z*-direction was applied to the free end of the nucleic acid (the other end was fixed to the origin), and no torsional constraint was applied to the system. The energy of the system due to the applied force was
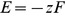
(2)Here *F* is the applied stretching force, and *z* is the helix extension. This simulation was equivalent to the measurement of force-extension curves in typical single-molecule magnetic tweezers or constant-force optical tweezers experiments [8], [13], [62], [84]–[86].

In the second type of simulation, the nucleic acid was subjected to a fixed stretching force and was required to maintain a link (which is equivalent to the bead rotation) close to a target value through a harmonic potential. The energy of the system was:
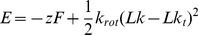
(3)Here *k_rot_* is the stiffness of the torsional trap (200 pN·nm by default), *Lk* is the helix link, and *Lk_t_* is the target link of the trap. This type of simulation corresponded to torsion-trapped tweezers experiments [14]–[17].

In both types of simulations, we computed the base-pair center and the coordinate frame of the terminal base-pair as well as the overall link of the helix after each full-helix MC update. The number of base pairs in the simulated double helices was set to 3,000 (3 kbp) in this work unless stated otherwise.

At the beginning of the simulation, we initialized the helix by assuming that all base-pair steps have step parameters equal to their average values in the input parameter database. We then performed by default 120 cycles of full-helix MC updates to relax the helix under the specified stretching force (but no link-constraint). For link-constrained simulations, we performed further relaxation steps analogous to the torsional trap experiments, which involve slowly rotating magnets of the torsional traps to bring the helix from zero-turn state to a highly twisted state. We first turned on the link constraint, but set initial target link equal to the current link of the helix. Then we performed the following cycles:

1. If the current target link was not within 20 degrees of the desired link (input by user), we changed the current target link by 20 degrees towards the desired link. Otherwise we set the target link to be the desired link and exited the loop.
2. We performed MC updates on the helix until the link of the helix was within 20 degrees of the current target link, then went back to step 1.

After this “trap-ramping” step, we further relaxed the helix under the specified force and link constraint for 50 cycles. These relaxation steps ensured that the state of the helix at the beginning of the simulation was random and representative of the specified force and link constraint, without memory of the initial conformation.

In the HelixMC package, all the parameters discussed above, including the number of base-pairs and the applied external forces and link constraint, can be modified by user inputs. The details of the setup of the HelixMC calculations reported in this work are given in Supplementary Methods. We set the number of samples collected during our simulations to ensure that the standard errors of the average extensions and links were below 0.2% (Table S12).

### Computation of link: From BPLM to ribbon model

Computing torsional properties and modeling torque in HelixMC required the integration of mathematical formulae developed in a number of separate papers by different authors. To document our final approach, we describe these equations and their connections here in some detail.

**Figure 6:**
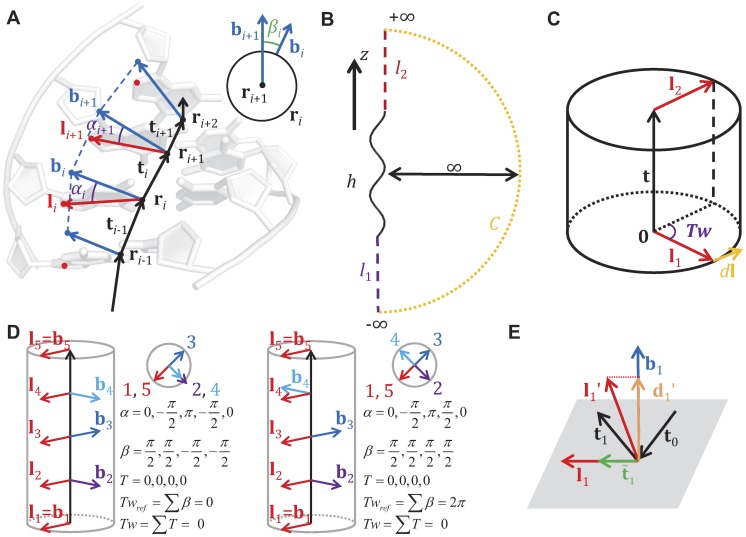
The base-pair level model and the twist and writhe calculation. (A) Illustration of the base-pair level model and ribbon model abstraction. Black dots and vectors: centers of base-pairs and the axis curve; red vectors: original ribbon vectors; blue vectors: reference ribbon vectors. See the main text for definition of *α* and *β*. (B) Conversion from an open curve to a closed curve for writhe calculation. (C) Twist for a straight line segment. (D) Effect of using reference ribbon vectors in the middle of a helix. The corresponding *α*, *β*, *T*, *Tw_ref_* and *Tw* are given. The original ribbon (red) is perfectly straight with zero twist. Using reference ribbon vectors either leads to no change (left) or a 2*π* difference (right) in twist. Taking *α* into account resolves the 2*π* difference. (E) Cases where the twist definition of Britton et al. [56] would fail to give reasonable answers.

The observed bead rotation in a single-molecule tweezers experiment is mathematically described by the link (also known as the linking number). The original definition for the link of circular dsDNA is based on a closed continuous ribbon model [87]–[91]. A ribbon is defined by two mathematical objects: an axis curve, which is a smooth non-self-intersecting closed curve following the axis of the polymer; and a set of ribbon vectors, which are unit normal vectors everywhere along the axis curve that are perpendicular to the axis curve and pointing to reference points on the polymer [91]. To compute the link, we followed previous work by Britton et al. [56] to convert the BPLM to a ribbon model (Fig. 6A). Here we defined the axis curve to be the line connecting the base-pair centers (black vectors, also known as the base-pair centerline), and the ribbon vectors to be the long-axis of the base-pair (red vectors). This discretization scheme leads to a polygonal axis curve where multiple straight lines are joined by sharp bends (at the base-pair centers), and the ribbon vectors are defined only at each bend. While this discretization is simple and easy to manipulate numerically, it leads to two problems that forbid direct applications of the formulations for the closed continuous ribbon model to the BPLM. First, the discretization leads to an axis curve with discontinuous first derivatives at each bend. Therefore the tangent vectors at these bends are ill-defined, and the corresponding ribbon vector is in general not perpendicular to both the axis curve segments connected to the bend. This behavior invalidates the original assumption that the axis curve is smooth and the ribbon vectors are always perpendicular to the axis curve. Second, the BPLM we studied here is for open duplexes, different from the closed curve assumption in the conventional treatment.

By the Călugăreanu theorem (also known as the White's formula, or the Călugăreanu-White-Fuller theorem), link equals the sum of writhe and twist [87]–[90]. Intuitively, writhe represents the degree of coiling of the ribbon axis curve, and twist represents the amount of internal twist stored in the ribbon due to the local rotations of ribbon vectors. The sum of coiling and internal twist gives the overall bead rotation of the ribbon. In the following sections, we discuss separately how to compute the writhe and twist for such an open, polygonal ribbon.

### Writhe calculation

Before discussing the writhe calculations for the BPLM, we first review the original definition of writhe, which described the coiling of the axis curve. The writhe of a smooth closed ribbon can be computed using the Gauss linking integral:
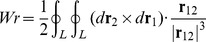
(4)Here **r**_1_ and **r**_2_ are the Cartesian coordinates of the axis curve, **r**_12_ = **r**_1_−**r**_2_ is a vector connecting points **r**_1_ and **r**_2_, and we compute writhe (and, below, link and twist) in units of radians. Note that writhe only depends on the axis curve of the ribbon. Fuller proposed a simplified version of this integral [91]:
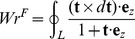
(5)Here **e** *_z_* is a unit vector aligned with *z*-axis, and **t** is the tangent vector of the axis curve. The Fuller writhe simplifies the original double integral into a single integral but is only correct modulo 4*π*.
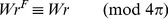
(6)Here the expression “*a*≡*b* (mod *n*)” means
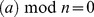
(7)Mathematically speaking, *a* and *b* are said to be congruent modulo *n*.

The calculation of writhe of BPLM in this work is based on previous studies on polygonal open curves [53]–[55]. In the section below, we will derive the formulas for computing writhe in BPLM, mainly following the approach developed by Rossetto and Maggs [54].

#### Constructing a closed curve

To apply Eqs. (4) and (5) to the BPLM, first we need to convert the open axis curve into a closed curve. This is achieved through the following steps (Fig. 6B) [54]. First, we attached two extension segments *l*_1_ and *l*_2_, to the lower and upper ends of the helix axis curve *h*. *l*_1_ and *l*_2_ are parallel to the *z*-axis, and are extended towards *z* = −*∞* and *z* = +*∞*. Second, we connect *l*_1_ and *l*_2_ with a curve *C*, such that *C*, *l*_1_ and *l*_2_ lie on the same plane. We also let *C* be far apart from the original axis curve, such that the distance between any point on *h* and any point on *C* approaches infinity. In this way, we can apply the above equations to a closed curve *L* = *h*+*l*_1_+*l*_2_+*C*. For Eq. (4), we get
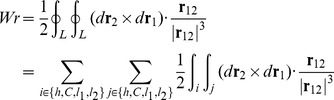
(8)Here 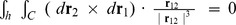, because any point in *C* and any point in *h* is infinitely distant away, therefore |r_12_| = *∞* and the integral vanishes. Terms that do not involve *h*, such as 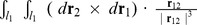 and 
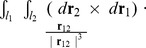
, also vanish, because *C*, *l*_1_ and *l*_2_ lie on the same plane, and therefore (*d* **r**_2_×*d* **r**_1_)· **r**_12_ = 0. Therefore we have
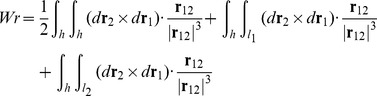
(9)Using a similar argument, we also have
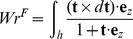
(10)


By the above construction, we extended the writhe definition for closed curves to open curves. In the next step, we will show how to evaluate Eq. (9) and (10) for a polygonal curve.

#### Evaluation of the Fuller writhe

We first compute the Fuller writhe (Eq. (10)) in our system. In this integral, the tangent vector **t** is a unit vector that starts as **e** *_z_* (tangent vector of *l*_1_), moves along the helix axis curve *h*, and ends up as **e**_z_ again (tangent vector of *l*_2_). If we translated the starting points of all the vectors **t** to the origin, **t** traces out a closed curve on a unit sphere, starting and ending at the zenith. Fuller proved that the spherical area enclosed by this closed curve equals the value of integral [91]. Based on this geometrical analogy, previous studies [54], [55] have shown that the spherical area enclosed by the tangent vectors of a polygonal line can be computed by breaking down the area into spherical triangles:
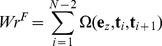
(11)Here Ω(**e** *_z_*, **t** *_i_*, **t** *_i_*_+1_) is the area (solid angle) of the spherical triangle with vertices defined by the three vectors, 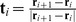 is each tangent vector, and *N* is the total number of base-pairs in the model. Note that Ω is a signed area, with sgn(Ω) = sgn((**e** *_z_*×**t** *_i_*) · **t** *_i_*_+1_). Here sgn is the sign function:
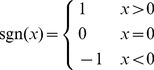
(12)The absolute value of the Ω equals the spherical excess of the triangle:
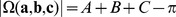
(13)Here *A*, *B*, *C* are the angles at the vertices a, b, c. These angles can be evaluated as
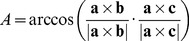
(14)
*B* and *C* can be evaluated in the same way.

#### Evaluation of the exact writhe

Now we evaluate the exact writhe formula (Eq. (9)). The first integral is a double integral involving only the helix axis curve *h*. This integral can be evaluated by noticing that it equals the spherical area swept by the unit vector 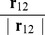. Similar to the above computation of Fuller writhe, we can break down this area for a polygonal line into spherical quadrangles [53]:
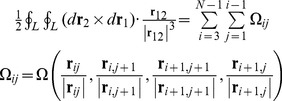
(15)Here Ω*_ij_* is the area of spherical quadrangle with vertices defined by the four unit vectors. Note that Ω*_ii_* = 0 and Ω*_i,i_*_+1_ = 0, therefore these terms are neglected from the summation. The quadrangle area can be computed similarly using its spherical excess:
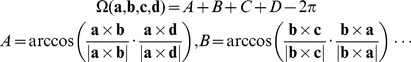
(16)And sgn(Ω*_ij_*) = sgn((**r** *_j,j+1_*×**r** *_i,i+1_*) · **r** *_ij_*).

To evaluate the second integral, we first translate our closed curve such that the lower end of *h* is at the origin. Because writhe is a geometrical property, it remains constant to such a translation. We call the new helix axis curve and extension segments *h*′, *l*_1_′ and *l*_2_′. In this new coordinate system, the segment *l*_1_′ overlaps with the −*z* axis, which simplifies the calculations. We now evaluate the integral:
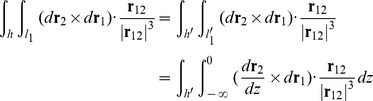
(17)Note here we let **r**_1_ to be the variable of outer integral and **r**_2_ to be the variable of inner integral. We know that 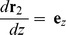 and **r**_2_ = (0, 0, *z*). We also let **r**_1_ = (*a*, *b*, *c*), where *a*, *b*, *c* can be any real number. For the inner integral in Eq. (17),
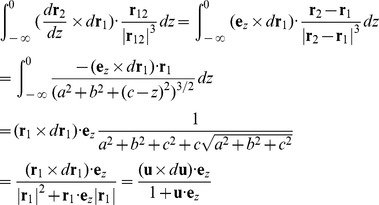
(18)Here we used the property (**e** *_z_*×*d* **r**_1_) · **r**_2_ = (**r**_2_×**e** *_z_*) · *d* **r**_1_ = 0. The integral in the second step can be computed by change of variables (with 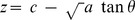). In the final step we let 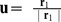. Combining Eq. (17) and (18)

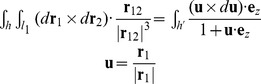
(19)Note this final expression is analogous to the Fuller writhe integral (Eq. (10)), therefore we can evaluate this discretized integral using the same algorithm. We can apply the same strategy to evaluate the third integral:
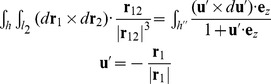
(20)Here *h*″ is the translated helix axis curve, such that the upper end of *h*″ is at the origin.

In the above paragraphs we demonstrated how to compute the Fuller writhe and the exact writhe for a polygonal open curve. The Fuller writhe involves computing a single sum, so the computational complexity is of *O(N)* (*N* is the number of base-pairs). However Fuller writhe is only correct modulo 4*π*, and it has been shown to give inaccurate results in low force and high torque situations ([54], [92] and results herein). On the other hand, the evaluation of the exact writhe involves computing a double sum, and is of *O(N^2^)*. Therefore it is currently difficult to perform link-constrained simulations for long helices using the exact writhe formula, where the link is evaluated in every update. In the section below, we will show that the Fuller formula can be used in link-constrained simulations as it gives correct answers in the force and link range used here. For simulations with low stretching force, however, the exact formula is needed to obtain accurate answer. Both writhe computation formulas are implemented in HelixMC. We note here that a sweep line algorithm may reduce the computational complexity to approximately *O(N log(N))* [93], but this algorithm is not yet implemented in HelixMC. Such accelerations will likely be necessary for HelixMC to model high link scenarios in which the Fuller formula breaks down due to, e.g., plectoneme formation.

### Twist calculation

The twist for a smooth ribbon can be computed as
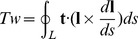
(21)Here **t** is the tangent vector of the axis curve, and **l** is the normalized ribbon vector. Unlike writhe, twist is a local identity, well defined on a curve segment of arbitrary length. Therefore twist is well defined for a smooth open curve. In addition, twist is additive. For our polygonal ribbon, the overall twist of the ribbon equals the sum of the twists of all the line segments. As an example, consider a straight line segment parallel to *z*-axis of length *L* (Fig. 6C). The ribbon vector starts as **l**_0_, varies smoothly and ends as **l**_1_. Using the fact that the tangent vector **t** = **e** *_z_* and the ribbon vectors are perpendicular to **t**, Eq. (21) can be evaluated as
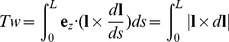
(22)Here we used the property that **l**×*d* **l** is parallel to **e** *_z_*. Geometrically, this integral is twice the area on unit circle swept by l throughout the integration. Therefore the twist of a straight line segment is just the angle (in radians) between the vectors **l**_0_ and **l**_1_. This result is consistent with the conventional definition of twist parameter in a base-pair step.

However, applying the above result for straight line segments to our polygonal ribbon is nontrivial, because here the ribbon vectors are not necessarily perpendicular to the straight line segments. A naïve strategy would be to simply sum the twist parameters of all base-pair steps in the helix to obtain the overall twist, but this sum turns out to be inconsistent with the ribbon twist considered in the Călugăreanu theorem. It thus cannot be added with writhe to produce a link that corresponds to the actual experimental observable of, e.g., bead rotation in a magnetic tweezers experiment. As pointed out by Britton and colleagues [56], the ribbon twist of dsDNA (‘twist’ discussed below refers to the ribbon twist, unless stated otherwise) is different from the conventional definition of twist parameter for a base-pair step, necessitating a new procedure to calculate twist for base-pair steps.

The main challenge in computing twist for the discrete chains of the nucleic acid helix is that the ribbon vector at each base pair, **l** *_i_*, is not in general, normal to the continuous axis curve traced by base pair centers **r** *_i_*, as is assumed in the mathematical treatment of ribbons. Our strategy therefore is to first define at each base pair a ‘reference’ ribbon vector b*_i_* that obeys this mathematical convention, and to compute a reference twist. We will then compute additional twist contributions by **l** *_i_* using its angle with b*_i_*. Fig. 6A illustrates the polygonal ribbon model. The choice of 
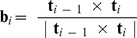
, where **t** *_i_*_−1_ and **t** *_i_* are unit vectors pointing into and out of **r** *_i_*, guarantees normality of **b** *_i_* to the axis curve. Then we can compute the reference twist based on the above result for straight line segments:
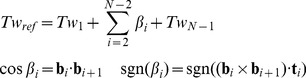
(23)Here *N* is the total number base-pairs in the model, *Tw*_1_ and *Tw_N_*_−1_ is the twist contribution of the first and the last base-pair steps (*N*−1 base-pair steps in total), where **b**'s are not defined. *β_i_* is the signed angle between the reference ribbon vector **b** *_i_* and **b** *_i+1_*. Note that because both **b** *_i_* and **b** *_i+1_* are orthogonal to **t** *_i_*, *β_i_* is also the dihedral angle **b** *_i_* - **t** *_i_* - **b** *_i+1_* (Fig. 6A, inset).

The use of alternative reference ribbon vectors to compute the twist can be justified with the following thought experiment. Imagine holding the two ends of a continuous ribbon, and then change the ribbon vectors by rotating the ribbon in the middle. As long as the two ends stay fixed, such changes of ribbon vectors do not affect the overall number of turns of the ribbon (i.e. the link). In addition, the writhe stays constant because it only depends on the axis curve, which is unmodified in this process. By the Călugăreanu theorem, we can conclude that the twist, which equals the link minus writhe, remains unchanged. Therefore in a continuous ribbon we may modify any ribbon vector except the two ends without affecting the overall twist. However for a discretized ribbon (as in our BPLM), such modifications of ribbon vectors may change the twist by 2*nπ*, where *n* is an integer (Fig. 6D). In general, we have the following modulo congruence relation between the true twist and reference twist (see Eq. (7) for definition of modulo congruence):
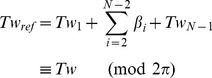
(24)To address the modulo 2*π* ambiguity, we must take into account whether the original ribbon vectors l*_i_* sweep out additional turns around the axis curve relative to the reference ribbon vectors b*_i_*. Here we calculate the local twist of each base-pair step as:
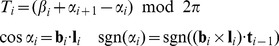
(25)Here *α_i_* is a signed angle between **l** *_i_* and **b** *_i_*; *T_i_* is folded into the range [−*π*, *π*) upon the modulo 2*π* operation. For the terminal base-pair steps, we first attach virtual segments to both ends, pointing towards −*z* and +*z* respectively, to obtain the corresponding **b** *_i_*, then Eq. (25) can be employed to compute *T*_1_ and *T_N_*_−1_ (illustrated in Fig. 6D). The overall twist can then be calculated by summing all the *T_i_*:
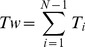
(26)


As an additional consistency check, Eq. (26) satisfies Eq. (24), as shown below.
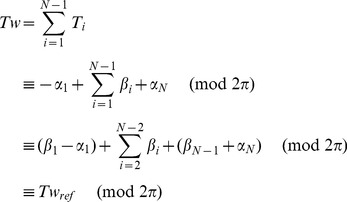
(27)The factors *α*_1_ and *α_N_* correspond to the twist contribution from two ends of the helix; all internal factors cancel.

Our twist definition is similar to the definition proposed by Britton and colleagues [56]. The main difference is in the definition of *α* angle. The previous proposal defined the tangent vector at base-pair *i* as 
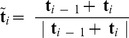
, then projected the original ribbon vector **l** *_i_* to the plane defined by 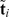, to obtain the new ribbon vector **d** *_i_*. Then *α* is defined as the angle between **d** *_i_* and **b** *_i_*:
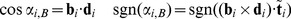
(28)While this definition is mathematically correct and gives results equivalent to our definition, the expression can become ill-defined when there is a sharp bend in the ribbon. Fig. 6E illustrates such cases. In the first example, the original ribbon vector **l**_1_ is parallel to the tangent vector 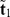, therefore the projection gives a null vector **d**_1_, making *α* ill-defined. In the second example, we take **l**_1_′ as the ribbon vector, then the projection gives **d**_1_′ parallel to **b**_1_, leading to a zero *α* even though **l**_1_′ and **b**_1_ are quite distinct from each other. In both cases, our definition just sets *α* equals to the angle between **l**_1_ and **b**_1_ and the angle between **l**_1_′ and **b**_1_, leading to a well-defined result. While this type of sharp bend does not occur in natural dsDNA, in our system the line segment of the last base-pair step and the added virtual segment can form such sharp bends, making the previous definition unsuitable for HelixMC.

The definition in Eq. (25) has two additional convenient properties. First, if the axis curve of the dsDNA/dsRNA is perfectly straight and pointing towards +*z*, and the base-pairs are all parallel to the *xy*-plane, the calculated ribbon twist equals to the sum of the base-step twist parameters [56]. Second, in our system setup, if the normal vector of the last base-pair aligns along +*z*, the computed link corresponds exactly to the bead rotation observed in single molecule tweezers experiments.

### Curation of the base-pair step parameters

**Figure 7:**
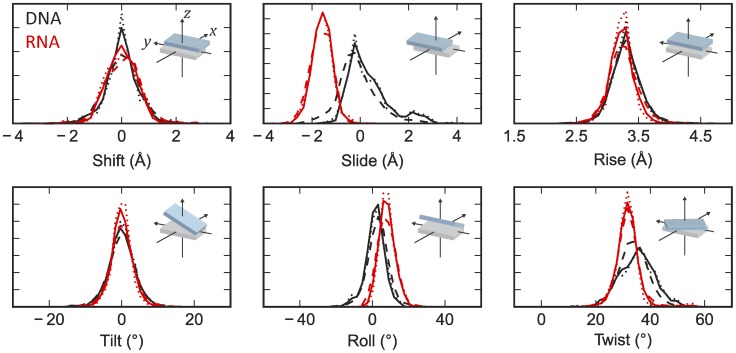
Normalized population distributions of the base-pair step parameters. Black: DNA. Red: RNA. Solid lines: default dataset (2.8 Å resolution cutoff, excluding protein-binding models). Dashed lines: 2.8_all dataset (2.8 Å resolution cutoff, including protein-binding models). Dotted lines: 2.0_noprot dataset (2.0 Å resolution cutoff, excluding protein-binding models). The inset illustrates the geometrical definition of each base-pair step parameter.

The multivariate Gaussian distributions for sampling are constructed using the base-pair step parameters from crystallographic models in the PDB. To ensure the quality of the data in the default parameter sets, we used models derived from data with resolutions better or equal to 2.8 Å. Protein-binding DNA/RNA structures were excluded from the dataset since protein binding may affect the deformability of the nucleic acids. We also tested several other selection schemes to estimate the systematic error, including using higher resolution cutoff (2.0 Å) or including protein-binding structures. We then used the 3DNA software [81], [82] to extract the base-pair step parameters for the canonical Watson-Crick base-pairs (i.e. not including G-U wobble base-pairs and other non-canonical base-pairs). Parameter sets with twist ≤5° (due to Z-DNA conformations), with rise ≥5.5 Å (due to ligand intercalation), or with any value more than four standard deviations away from the mean were discarded as outliers. For the dsDNA datasets, we noticed that there were two major clusters in the data, corresponding to the A-form and B-form dsDNA (except for the ‘DNA_2.8_all’ dataset, where the protein binding rendered A-DNA and B-DNA inseparable by clustering). We used the k-means algorithm to separate the two clusters and only used the B-DNA parameters in sampling (Table 1, Supplementary Methods and Fig. S7). For Z-DNA, the dataset is composed of two distinct base-pair step distribution for the GC steps and the CG steps. The population distribution for the Z-DNA dataset is shown in Fig. S8. Detailed statistics and population distribution of the curated dataset are shown in Table 1, S13 and Fig. 7. Fig. S9 shows the correlation plots between each base-pair step parameter for the default dataset.

For each type of base-pair step (16 in total, e.g. 5′-AT-3′/5′-AT-3′, 5′-CA-3′/5′-TG-3′, etc.), a multivariate Gaussian was fitted based on the corresponding six base-pair step parameters, enabling sequence-dependent simulations. We note here that one can also categorize the base-pair parameters into 10 independent sequence-specific categories using the symmetry of the base-pair steps [40], [80]. This symmetrization is not the default option in HelixMC; symmetrization gives minor changes in the predicted mechanical properties (Supplementary Methods and Table S2).

For simulations with random sequence, in each update we randomly picked a distribution from the 16 types of step parameters and drew samples from it. In this sampling scheme, we effectively averaged the 16 types of parameters, so all base-pair steps follow the same parameter distribution [40]. The distribution can be further approximated with a single multivariate Gaussian (Supplementary Results). The approximation leads to a reduced number of parameters in the model, and therefore facilitates the understanding the effect of each parameter on the observed mechanical properties. However, we note that this sampling scheme may lead to unrealistic base-pair step combinations (for example, 5′-AT-3′/5′-AT-3′ followed by 5′-GC-3′/5′-GC-3′), therefore the sequence of the RNA is not always well defined in each simulation snapshot. To justify that our sampling scheme indeed gave reasonable estimates of the mechanical properties of a random RNA, we also performed simulations with a single randomly generated RNA sequence (Table S14). The obtained mechanical properties using a single random sequence agreed within simulation error to our default random sequence simulation.

In addition, we observed that some of the population distributions in our dataset did not appear Gaussian (Fig. 7). To test the validity of the Gaussian approximation, we also tested a different sampling scheme, by randomly picking parameter sets existing in the database without assuming Gaussianity, and obtained nearly undistinguishable results (see Results). All the curated parameter sets and sampling schemes used in this work are available and further documented in the HelixMC package.

### Software availability

The bottleneck steps of HelixMC have been optimized in C (using Cython); a typical single-point HelixMC calculation for a DNA/RNA helix of experimental length (few kilo-base-pairs) takes minutes to hours on a standard desktop computer (Table S15). HelixMC is coded in Python in an object-oriented fashion that allows easy modification and extension, is free and open-source (http://github.com/fcchou/helixmc), and enables fast and accurate predictions with available computational power.

### Validation of the link calculations

**Figure 8:**
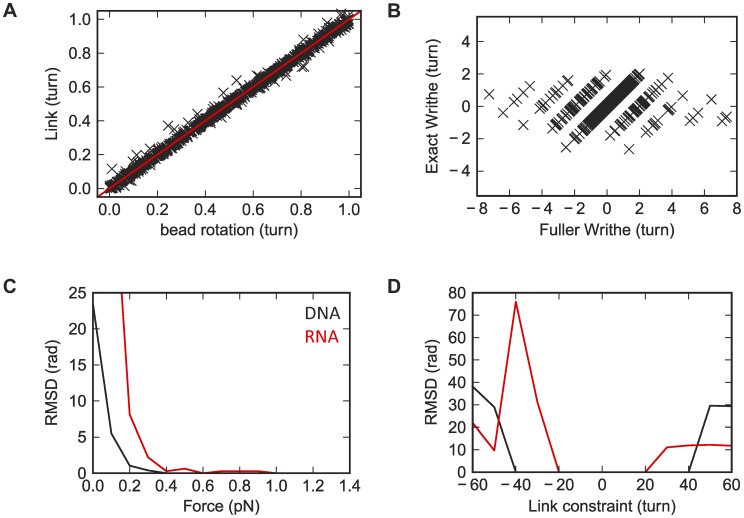
Validation of the link calculations. (A) Plot of calculated link vs. bead rotation in DNA simulation with 7 pN stretching force and no link constraint. The red line is the function y = x. The link is folded into the range of 0 to 1 turn for better comparison. RMSD of the calculated link is 5.7°. (B) Plot of Fuller writhe vs. exact writhe in DNA simulation with 0.1 pN stretching force and no link constraint. The separation of two turns between parallel traces demonstrates that Fuller writhe is only correct modulo 4π in this criteria. (C) RMSD for Fuller writhe at different stretching forces with no link constraint. Black: DNA; Red: dsRNA. (D) RMSD for Fuller writhe at different link constraints in simulations at 7 pN stretching force.

We numerically tested the validity of the link calculations above by comparing simulated link values to the bead rotations (Fig. 8A). Here the “bead rotation” is defined as the angle between the *y*-axis of the global coordinate and the projection of the ribbon vector of the last base-pair to the *xy*-plane. This is equivalent to attaching a virtual bead along the ribbon vector of the last base-pair and observing its rotation, analogous to recent single-molecule tweezers experiments [17]. For better comparison, in Fig. 8A we folded the computed link into the range of [−*π*, *π*), and found that the experimentally observed bead-rotation indeed corresponds to the link. The match between the link and the bead rotation was close but not exact (RMSD of 4.5°), because the normal vector of the last base-pair did not point exactly to +*z* during the simulation; this discrepancy induces negligible error in computed helix mechanical properties.

As discussed above, the Fuller formula is faster but gives exact writhes only in certain conditions. The formula breaks down if the helix path fluctuates so that segments point away from the applied force (towards −*z*). Fig. 8B shows a plot of exact writhe vs. Fuller writhe in a simulation of 3 kbp dsDNA at 0.1 pN stretching force. It is apparent that in this setting the Fuller formula is only correct modulo 4*π* (two turns; spacing between parallel lines). To test under which conditions the Fuller formula was accurate, we computed its RMSD error to exact writhe across simulations. For force-extension simulations, Fuller writhe is effectively exact if the force is larger than 0.4 pN for dsDNA and 1 pN for dsRNA (Fig. 8C). For link-constrained simulations, the Fuller formula holds in the current simulated link-range, but breaks down when the target link exceeds ±40 turns for DNA and ±20 turns for dsRNA (corresponding to supercoiling densities of 0.022 and 0.012; Fig. 8D).

### Fitting model for simulated and experimental data

As with experimental measurements, the simulated data were summarized through fits to the elastic rod model, which assume that the total energy of the helix without external force and torque can be expressed using the above parameters by an integral along the helix axis curve *s*:
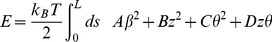
(29)Here *L* is the helix contour length, *k_B_* is the Boltzmann constant and *T* is the temperature. The constants are bending persistence length *A*, *B = S/k_B_T* the stretching stiffness (where *S* is the stretch modulus), torsional persistence length *C*, and *D = g/k_B_T* is the unit-less link-extension coupling (here we use the convention in ref. [14], where *g* has units of pN·nm). The three quantities *β*, *z* and *θ* describe the deformations per unit length of a short rod segment. *β* is the bending deformation that measures how the tangent vector changes along the rod, *z* is the extensional deformation that measures the change in the length of the segment, and *θ* is the torsional deformation that determines how the each segment is rotated around the rod axis with respect to adjacent segment. The analytical equations used to fit experimental measurements, derived from this model, are compiled in the Supplementary Methods.

## Supporting Information

Figure S1 **Comparison of extensive WLC model by Bouchiat et al. and Odijk**. Blue lines: fits of the model by Bouchiat et al. Red broken lines: fits of Odijk WLC model. (A) DNA. (B) RNA.
(EPS)

Figure S2
**Effect of xy-constraint on the mechanical behavior for dsDNA and dsRNA**. Black: without xy-constraints (regular behavior); Red: with xy-constraints. (A, C, E) DNA; (B, D, F) RNA. (A, B) force-extension; (C, D) effective torsional persistence length vs. force; (E, F) link vs. force. The constraint is a harmonic constraint centered at *x* = *y* = 0 (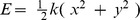, *x* and *y* is the corresponding coordinate components for the last base-pair in the helix). The force constant *k* is set to 0.0025 pN/Å.(EPS)

Figure S3 **Plots of the helix extension (end-to-end distances) vs. the stretching force**. (A) DNA; (B) RNA. The blue lines are linear fits using the last three data points. For both DNA and RNA the linear relationship holds only when force >15 pN.
(EPS)

Figure S4 **Link-extension couplings computed using simulations of two different experimental setups**. Here g_1_ is computed from the slopes of link vs. force plots; g_2_ are computed from the slopes of extension vs. link constraint plots. The data can be well fitted by a straight line (R^2^ = 0.96).
(EPS)

Figure S5 **Effect of multi-base-pair parameter sets on the mechanical properties of dsDNA**. Red: multi-base-pair parameter sets. Black: control parameter sets. (A) Bending persistence length; (B) Stretch modulus; (C) Torsional persistence length; (D) Link-extension coupling (slopes of link vs. force measurements).
(EPS)

Figure S6 **Effect of multi-base-pair parameter sets on the mechanical properties of dsRNA**. Red: multi-base-pair parameter sets. Black: control parameter sets. (A) Bending persistence length; (B) Stretch modulus; (C) Torsional persistence length; (D) Link-extension coupling (slopes of link vs. force measurements).
(EPS)

Figure S7 **Population distributions of base-pair step parameters during k-mean clustering**. The clustering is used in curating the “DNA_default” parameter set. The black line represents the original full data extracted from the PDB. This full data is clustered into 3 different clusters represented by the blue, green, and red lines. Red and green clusters represent B-DNA and are retained; blue cluster is removed from the final dataset as it represents A-DNA conformations.
(EPS)

Figure S8 **Population distribution of base-pair step parameters for Z-DNA**. The black lines represent the GC/GC base-pair steps and red lines represent the CG/CG steps.
(EPS)

Figure S9 **The correlations between the base-pair step parameters**. Black: DNA; Red: RNA. The ellipses represent the contours of the multivariate Gaussian at three standard deviations.
(EPS)

Table S1 **Comparison of bending persistence length (in nm) computed by different methods**. The values in parenthesis are the corresponding fitting errors. See Table 1 for detailed description for each parameter set.
(DOC)

Table S2 **Effect of the symmetrization of the base-pair step parameter set on predicted mechanical properties**. The values in parenthesis are the corresponding fitting errors. See Table 1 for detailed description for each parameter set.
(DOC)

Table S3 **Comparison of stretch modulus (in pN) computed by different methods**. The values in parenthesis are the corresponding fitting errors. See Table 1 for detailed description for each parameter set.
(DOC)

Table S4 **Effect of parameter grafting in the predicted mechanical properties**. ^1^Single Gaussian parameter set built from the default dataset. ^2^Chimera single Gaussian parameter set. See Supplementary Results for explanation.
(DOC)

Table S5
**Changes of conformational parameters upon stretching for 100-bp DNA and RNA helices**. Simulations are performed using the default parameter set. The changes of shift, slide and tilt upon stretching are small (below 0.02 standard deviation) and therefore not shown. ^1^ 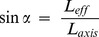, where *L_axis_* is the length of the axis curve, *L_eff_* is the effective helix contour length and *α* is the super-helical pitch angle. 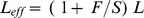, where *F* is the applied stretching force, *S* is the stretch modulus, and *L* is the helix contour length. See Supplementary Methods for more information. ^2^ The first value is the average parameter, followed by the corresponding Z-score.(DOC)

Table S6 **Steric clashes in simulations**. Simulations are performed on helices of 3,000 base-pairs. In each simulation, 1,000 frames are generated for checking the number of steric clashed conformations.
(DOC)

Table S7 **Comparison of torsional persistence length (in nm) computed by different methods**. The values in parenthesis are the corresponding fitting errors. See Table 1 for detailed description for each parameter set.
(DOC)

Table S8 **Effect of individual parameters in covariance matrix for DNA**. ^1^ Values for the original parameter set computed using the full simulation (see the ‘DNA_gau’ entry in Table 1). ^2^ Halving or doubling the variance of ‘shift’ parameter in the covariance matrix. ^3^ Reverse the sign of the shift-slide covariance in the covariance matrix.
(DOC)

Table S9 **Effect of individual parameters in covariance matrix for RNA**. See captions of Table S9 for detailed explanations.
(DOC)

Table S10 **Standard deviations of parameters for random DNA, poly(A)/poly(T) and Z-DNA**. ^1^ Z-DNA has a minimum repetitive unit of two base-pairs, therefore it has two distinct step parameter set (GC and CG).
(DOC)

Table S11 **Acceptance rate of the Monte Carlo simulations**. Link constrained simulations are performed at 7 pN stretching force.
(DOC)

Table S12 **Values and sampling error of observables in HelixMC simulations at difference forces and link-constraints**. The values in parenthesis are the corresponding sampling errors.
(DOC)

Table S13 **Covariance matrices for DNA and RNA default parameter sets**. Data are derived from crystallographic models excluding proteins and with diffraction resolutions of 2.8 Å or better. Additional data sets, including sequence-dependent parameters, are available in the HelixMC package (http://github.com/fcchou/helixmc).
(DOC)

Table S14 **Comparison of simulations with default random sequence and a single random sequence**.
(DOC)

Table S15 **Example computational time of HelixMC on a Linux desktop**. Computer specification: Intel Core i7-3770 CPU @ 3.40 GHz, 24 GB RAM. Operation system: Linux Mint 13 Maya. Computation is performed using Enthought Python Distribution 7.3-1 academic edition. Each simulation uses a single thread and ∼700 MB of memory. All simulations are performed with 7 pN stretching force.
(DOC)

Text S1 **Supplementary Methods**.
(DOC)

Text S2 **Supplementary Results**.
(DOC)
