# Supplementary material for: Blind Predictions of DNA and RNA Tweezers Experiments with Force and Torque
Source: PLoS Comput Biol. 2014 Aug 7;10(8):e1003756. doi: 10.1371/journal.pcbi.1003756 (PMC4125081; doi:10.1371/journal.pcbi.1003756)
Supplement: Table S1 — Comparison of bending persistence length (in nm) computed by different methods. The values in parenthesis are the corresponding fitting errors. See Table 1 for detailed description for each parameter set. (DOC) [file pcbi.1003756.s010.doc]

Table S1. Comparison of bending persistence length (in nm) computed by different methods.

| Simulations^1^ | | Extensible WLC | Inextensible WLC | Odijk WLC | Olson’s matrix averaging |
| --- | --- | --- | --- | --- | --- |
| D N A | default | 54.7(0.6) | 53.1(1.0) | 54.5(1.0) | 53.0(0.1) |
|  | default_frag | 54.5(0.6) | 52.9(1.0) | 54.3(0.9) | 53.5(0.1) |
|  | 2.8_all | 39.4(0.5) | 38.2(0.8) | 41.9(1.4) | 38.2(0.1) |
|  | 2.8_all_frag | 39.1(0.4) | 37.9(0.8) | 41.5(1.3) | 38.3(0.1) |
|  | 2.0_noprot | 50.0(0.5) | 48.7(0.8) | 50.4(0.9) | 48.7(0.1) |
|  | 2.0_noprot_frag | 50.0(0.6) | 48.7(1.0) | 50.7(1.1) | 49.8(0.1) |
|  | poly(A)/poly(T) default | 111.1(0.8) | 108.9(1.3) | 107.3(0.3) | 108.8(0.1) |
|  | poly(A)/poly(T) 2.8_all | 38.5(0.4) | 37.7 (0.6) | 41.0(1.4) | 37.2(0.1) |
|  | poly(G)/poly(C) default | 62.0(0.6) | 59.4(1.4) | 60.8(0.7) | 59.8(0.1) |
|  | poly(G)/poly(C) 2.8_all | 51.5(0.6) | 49.3(1.2) | 51.7(1.0) | 49.0(0.1) |
|  | Z-DNA | 175.4(1.0) | 167.5(3.3) | 172.7(0.4) | 170.9(0.2) |
| R N A | default | 66.3(0.9) | 61.8(2.1) | 65.3(0.7) | 62.9(0.1) |
|  | default_frag | 66.5(0.8) | 62.0(2.0) | 65.1(0.8) | 63.4(0.1) |
|  | 2.8_all | 46.9(0.7) | 43.6(1.6) | 48.2(1.2) | 44.3(0.1) |
|  | 2.8_all_frag | 46.9(0.7) | 43.7(1.6) | 48.2(1.2) | 44.4(0.1) |
|  | 2.0_noprot | 76.3(1.1) | 70.6(2.6) | 74.6(0.6) | 72.6(0.1) |
|  | 2.0_noprot_frag | 75.8(0.9) | 70.5(2.4) | 74.0(0.7) | 73.4(0.1) |
|  | poly(A)/poly(U) default | 82.0(0.9) | 77.4(2.2) | 79.8(0.5) | 78.2(0.1) |
|  | poly(A)/poly(U) 2.8_all | 59.1(0.8) | 55.6(1.7) | 58.8(0.9) | 55.9(0.1) |
|  | poly(G)/poly(C) default | 86.4(0.9) | 80.8(2.5) | 84.1(0.4) | 82.7(0.1) |
|  | poly(G)/poly(C) 2.8_all | 51.7(0.7) | 47.5(1.9) | 52.2(1.1) | 59.8(0.1) |

The values in parenthesis are the corresponding fitting errors. See Table 1 for detailed description for each parameter set.
