# Supplementary material for: Blind Predictions of DNA and RNA Tweezers Experiments with Force and Torque
Source: PLoS Comput Biol. 2014 Aug 7;10(8):e1003756. doi: 10.1371/journal.pcbi.1003756 (PMC4125081; doi:10.1371/journal.pcbi.1003756)
Supplement: Table S2 — Effect of the symmetrization of the base-pair step parameter set on predicted mechanical properties. The values in parenthesis are the corresponding fitting errors. See Table 1 for detailed description for each parameter set. (DOC) [file pcbi.1003756.s011.doc]

Table S2. Effect of the symmetrization of the base-pair step parameter set on predicted mechanical properties.

| Simulations | | *A*: bending persistence  (nm) | *S*: stretch modulus S(pN) | *C*: torsional persistence length(nm) | Slope of link vs. force (rad/pN) | Slope of extension vs. link (nm/turn) | *g*: link-extension coupling 1 (pN·nm) | *g*: link-extension coupling 2 (pN·nm) |
| --- | --- | --- | --- | --- | --- | --- | --- | --- |
| DNA | default | 54.7(0.6) | 1956.9(102.1) | 28.8(0.1) | 0.202(0.001) | 0.473(0.015) | −131.0(7.5) | −147.4(9.0) |
|  | default symmetrized | 55.1(0.6) | 1971.0(114.0) | 28.8(0.3) | 0.202(0.001) | 0.465(0.014) | −131.8(8.4) | −145.7(9.5) |
|  | poly(A)/poly(T) 2.8_all | 38.5(0.4) | 2403.1(170.3) | 38.1(0.1) | 0.086(0.004) | 0.351(0.014) | −97.2(8.2) | −134.3(11.0) |
|  | poly(A)/poly(T) 2.8_all symmetrized | 44.5(0.5) | 2525.4(170.7) | 47.5(0.3) | 0.073(0.002) | 0.330(0.020) | −108.3(8.3) | −132.4(11.9) |
| RNA | default | 66.3(0.9) | 979.0(40.5) | 53.0(0.2) | 0.161(0.001) | 0.797(0.011) | −116.5(5.2) | −124.2(5.4) |
|  | default symmetrized | 66.2(0.9) | 990.6(41.2) | 53.3(0.2) | 0.160(0.001) | 0.811(0.014) | −117.8(5.3) | −127.9(5.8) |
|  | poly(A)/poly(T) 2.8_all | 59.1(0.8) | 1049.2(48.5) | 69.2(0.3) | 0.145(0.001) | 0.981(0.026) | −146.7(7.5) | −163.9(8.8) |
|  | poly(A)/poly(T) 2.8_all symmetrized | 71.6(1.0) | 1208.4(56.7) | 62.0(0.1) | 0.175(0.001) | 1.046(0.017) | −175.6(9.2) | −201.1(10.0) |

The values in parenthesis are the corresponding fitting errors. See Table 1 for detailed description for each parameter set.
