# Supplementary material for: Blind Predictions of DNA and RNA Tweezers Experiments with Force and Torque
Source: PLoS Comput Biol. 2014 Aug 7;10(8):e1003756. doi: 10.1371/journal.pcbi.1003756 (PMC4125081; doi:10.1371/journal.pcbi.1003756)
Supplement: Table S3 — Comparison of stretch modulus (in pN) computed by different methods. The values in parenthesis are the corresponding fitting errors. See Table 1 for detailed description for each parameter set. (DOC) [file pcbi.1003756.s012.doc]

Table S3. Comparison of stretch modulus (in pN) computed by different methods.

| Simulations^1^ | | Extensible WLC | Odijk WLC |
| --- | --- | --- | --- |
| D N A | default | 1956.9(102.1) | 1592.2(539.3) |
|  | default_frag | 1993.4(106.1) | 1596.8(504.2) |
|  | 2.8_all | 1504.8(90.8) | 903.0(378.7) |
|  | 2.8_all_frag | 1517.8(78.4) | 922.0(390.2) |
|  | 2.0_noprot | 2146.7(115.2) | 1572.7(579.4) |
|  | 2.0_noprot_frag | 2122.9(146.0) | 1473.5(585.8) |
|  | poly(A)/poly(T) default | 4373.4(190.8) | 5140.3(560.9) |
|  | poly(A)/poly(T) 2.8_all | 2403.1(170.3) | 1139.5(623.9) |
|  | poly(G)/poly(C) default | 1500.3(59.5) | 1409.7(257.0) |
|  | poly(G)/poly(C) 2.8_all | 1315.1(57.3) | 1100.0(284.3) |
|  | Z-DNA | 2618.5(59.0) | 2674.8(95.1) |
| R N A | default | 979.0(40.5) | 939.1(105.8) |
|  | default_frag | 983.1(37.9) | 958.9(116.2) |
|  | 2.8_all | 776.7(36.5) | 651.4(140.4) |
|  | 2.8_all_frag | 773.1(35.7) | 651.6(136.8) |
|  | 2.0_noprot | 996.9(42.2) | 998.9(80.6) |
|  | 2.0_noprot_frag | 1034.0(39.3) | 1033.8(95.0) |
|  | poly(A)/poly(U) default | 1354.0(51.3) | 1361.7(114.1) |
|  | poly(A)/poly(U) 2.8_all | 1049.1(48.5) | 949.2(154.4) |
|  | poly(G)/poly(C) default | 1197.1(41.7) | 1214.8(58.4) |
|  | poly(G)/poly(C) 2.8_all | 706.2(29.4) | 636.7(104.8) |

The values in parenthesis are the corresponding fitting errors. See Table 1 for detailed description for each parameter set.
