# Supplementary material for: Blind Predictions of DNA and RNA Tweezers Experiments with Force and Torque
Source: PLoS Comput Biol. 2014 Aug 7;10(8):e1003756. doi: 10.1371/journal.pcbi.1003756 (PMC4125081; doi:10.1371/journal.pcbi.1003756)
Supplement: Table S4 — Effect of parameter grafting in the predicted mechanical properties. 1Single Gaussian parameter set built from the default dataset. 2Chimera single Gaussian parameter set. See Supplementary Results for explanation. (DOC) [file pcbi.1003756.s013.doc]

Table S4. Effect of parameter grafting in the predicted mechanical properties.

| Simulations | *A*: bending persistence  (nm) | *S*: stretch modulus S(pN) | *C*: torsional persistence length(nm) | Slope of link vs. force (rad/pN) | Slope of extension vs. link (nm/turn) | *g*: link-extension coupling 1 (pN·nm) | *g*: link-extension coupling 2 (pN·nm) |
| --- | --- | --- | --- | --- | --- | --- | --- |
| DNA_gau^1^ | 54.6(0.6) | 1907.7(95.9) | 28.5(0.1) | 0.216(0.001) | 0.494(0.014) | −134.0(7.4) | −150.0(8.6) |
| RNA_gau | 66.3(0.9) | 964.8(40.6) | 53.2(0.1) | 0.162(0.002) | 0.841(0.012) | −116.0(5.4) | −129.1(5.7) |
| DNA_gau_graft^2^ | 81.9(0.7) | 2824.1(121.5) | 60.7(0.2) | 0.054(0.000) | 0.237(0.015) | −113.7(5.0) | −106.7(8.1) |
| RNA_gau_graft | 39.4(0.8) | 425.7(22.3) | 28.4(0.1) | 0.602(0.011) | 1.774(0.013) | −90.3(6.0) | −120.2(6.3) |

^1^Single Gaussian parameter set built from the default dataset.

^2^Chimera single Gaussian parameter set. See Supplementary Results for explanation.
