# Supplementary material for: Blind Predictions of DNA and RNA Tweezers Experiments with Force and Torque
Source: PLoS Comput Biol. 2014 Aug 7;10(8):e1003756. doi: 10.1371/journal.pcbi.1003756 (PMC4125081; doi:10.1371/journal.pcbi.1003756)
Supplement: Table S5 — Changes of conformational parameters upon stretching for 100-bp DNA and RNA helices. Simulations are performed using the default parameter set. The changes of shift, slide and tilt upon stretching are small (below 0.02 standard deviation) and therefore not shown. 1 , where Laxis is the length of the axis curve, Leff is the effective helix contour length and α is the super-helical pitch angle. , where F is the applied stretching force, S is the stretch modulus, and L is the helix contour length. See Supplementary Methods for more information. 2 The first value is the average parameter, followed by the corresponding Z-score. (DOC) [file pcbi.1003756.s014.doc]

Table S5. Changes of conformational parameters upon stretching for 100-bp DNA and RNA helices.

|  | Force (pN) | Avg. extension (nm) | Avg. sin*α*^1^ | Avg. rise (Å)^2^ | Avg. roll (°) | Avg. twist (°) |
| --- | --- | --- | --- | --- | --- | --- |
| D N A | 1 | 28.5 | 0.957 | 3.300 / 0.006 | 1.56 / −0.001 | 35.22 / 0.004 |
|  | 5 | 30.9 | 0.957 | 3.305 / 0.020 | 1.56 / −0.007 | 35.27 / 0.019 |
|  | 10 | 31.6 | 0.968 | 3.310 / 0.036 | 1.57 / −0.005 | 35.33 / 0.039 |
|  | 20 | 32.2 | 0.959 | 3.323 / 0.075 | 1.56 / −0.007 | 35.45 / 0.076 |
|  | 40 | 32.8 | 0.962 | 3.349 / 0.153 | 1.55 / −0.009 | 35.69 / 0.152 |
| R N A | 1 | 24.0 | 0.758 | 3.225 / 0.002 | 7.85 / −0.008 | 31.74 / 0.008 |
|  | 5 | 25.9 | 0.761 | 3.228 / 0.012 | 7.75 / −0.027 | 31.80 / 0.029 |
|  | 10 | 26.6 | 0.764 | 3.230 / 0.021 | 7.62 / −0.055 | 31.88 / 0.061 |
|  | 20 | 27.2 | 0.771 | 3.237 / 0.045 | 7.36 / −0.108 | 32.03 / 0.115 |
|  | 40 | 27.9 | 0.784 | 3.250 / 0.093 | 6.90 / −0.201 | 32.33 / 0.226 |

Simulations are performed using the default parameter set. The changes of shift, slide and tilt upon stretching are small (below 0.02 standard deviation) and therefore not shown.

^1^$\sin\alpha=\frac{L_{eff}}{L_{axis}}$, where *L_axis_* is the length of the axis curve, *L_eff_* is the effective helix contour length and *α* is the super-helical pitch angle. $L_{eff}=(1+F/S)L$, where *F* is the applied stretching force, *S* is the stretch modulus, and *L* is the helix contour length. See Supplementary Methods for more information.

^2^ The first value is the average parameter, followed by the corresponding Z-score.
