# Supplementary material for: Blind Predictions of DNA and RNA Tweezers Experiments with Force and Torque
Source: PLoS Comput Biol. 2014 Aug 7;10(8):e1003756. doi: 10.1371/journal.pcbi.1003756 (PMC4125081; doi:10.1371/journal.pcbi.1003756)
Supplement: Table S6 — Steric clashes in simulations. Simulations are performed on helices of 3,000 base-pairs. In each simulation, 1,000 frames are generated for checking the number of steric clashed conformations. (DOC) [file pcbi.1003756.s015.doc]

Table S6. Steric clashes in simulations.

|  | DNA | | RNA | |
| --- | --- | --- | --- | --- |
| Force (pN) | 0 | 0.4 | 0 | 0.4 |
| Steric Clashes | 56 / 1000 | 0 / 1000 | 28 / 1000 | 0 / 1000 |

Simulations are performed on helices of 3,000 base-pairs. In each simulation, 1,000 frames are generated for checking the number of steric clashed conformations.
