# Supplementary material for: Blind Predictions of DNA and RNA Tweezers Experiments with Force and Torque
Source: PLoS Comput Biol. 2014 Aug 7;10(8):e1003756. doi: 10.1371/journal.pcbi.1003756 (PMC4125081; doi:10.1371/journal.pcbi.1003756)
Supplement: Table S7 — Comparison of torsional persistence length (in nm) computed by different methods. The values in parenthesis are the corresponding fitting errors. See Table 1 for detailed description for each parameter set. (DOC) [file pcbi.1003756.s016.doc]

Table S7. Comparison of torsional persistence length (in nm) computed by different methods.

| Simulations^1^ | | Effective torsional persistence vs. force | Torque vs.  constrained link |
| --- | --- | --- | --- |
| D N A | default | 28.8(0.1) | 28.4(0.2) |
|  | default_frag | 28.7(0.1) | 28.2(0.2) |
|  | 2.8_all | 40.5(0.3) | 38.9(0.3) |
|  | 2.8_all_frag | 40.4(0.3) | 38.9(0.3) |
|  | 2.0_noprot | 27.0(0.1) | 26.8(0.2) |
|  | 2.0_noprot_frag | 27.0(0.2) | 26.9(0.3) |
|  | poly(A)/poly(T) default | 97.7(0.4) | 94.8(0.1) |
|  | poly(A)/poly(T) 2.8_all | 38.1(0.1) | 37.6(0.2) |
|  | poly(G)/poly(C) default | 28.9(0.2) | 28.3(0.2) |
|  | poly(G)/poly(C) 2.8_all | 53.7(0.3) | 52.4(0.2) |
|  | Z-DNA | 126.9(0.3) | 125.1(0.1) |
| R N A | Default | 53.0(0.2) | 51.0(0.2) |
|  | default_frag | 52.7(0.2) | 51.1(0.2) |
|  | 2.8_all | 42.4(0.3) | 41.1(0.2) |
|  | 2.8_all_frag | 42.8(0.3) | 41.0(0.2) |
|  | 2.0_noprot | 49.7(0.2) | 47.4(0.4) |
|  | 2.0_noprot_frag | 49.9(0.2) | 47.4(0.5) |
|  | poly(A)/poly(U) default | 57.6(0.3) | 55.8(0.1) |
|  | poly(A)/poly(U) 2.8_all | 69.2(0.3) | 68.1(0.1) |
|  | poly(G)/poly(C) default | 62.9(0.4) | 61.4(0.2) |
|  | poly(G)/poly(C) 2.8_all | 34.8(0.1) | 34.0(0.2) |

The values in parenthesis are the corresponding fitting errors. See Table 1 for detailed description for each parameter set.
