# Supplementary material for: Blind Predictions of DNA and RNA Tweezers Experiments with Force and Torque
Source: PLoS Comput Biol. 2014 Aug 7;10(8):e1003756. doi: 10.1371/journal.pcbi.1003756 (PMC4125081; doi:10.1371/journal.pcbi.1003756)
Supplement: Table S8 — Effect of individual parameters in covariance matrix for DNA. 1 Values for the original parameter set computed using the full simulation (see the ‘DNA_gau’ entry in Table 1). 2 Halving or doubling the variance of ‘shift’ parameter in the covariance matrix. 3 Reverse the sign of the shift-slide covariance in the covariance matrix. (DOC) [file pcbi.1003756.s017.doc]

Table S8. Effect of individual parameters in covariance matrix for DNA.

|  | Bending persistence length (nm) | Change (%) | Stretch modulus (pN) | Change (%) | Torsional Persistence length (nm) | Change (%) | Link vs. force slope (rad/pN) | Change (%) |
| --- | --- | --- | --- | --- | --- | --- | --- | --- |
| Full^1^ | 54.6 |  | 1908 |  | 28.0 |  | 0.216 |  |
| Original | 53.0 |  | 1876 | 0.0 | 29.0 |  | 0.226 |  |
| Shift_half^2^ | 53.0 | 0.1 | 1880 | 0.2 | 28.7 | −1.0 | 0.224 | −1.1 |
| Shift_double^2^ | 53.1 | 0.1 | 1683 | −10.3 | 28.1 | −3.0 | 0.209 | −7.5 |
| Slide_half | 53.0 | 0.0 | 2112 | 12.6 | 28.7 | −1.0 | 0.246 | 8.9 |
| Slide_double | 53.0 | 0.0 | 1379 | −26.5 | 32.0 | 10.3 | 0.200 | −11.6 |
| Rise_half | 53.0 | 0.0 | 3028 | 61.4 | 30.3 | 4.5 | 0.239 | 5.8 |
| Rise_double | 53.0 | 0.1 | 1089 | −42.0 | 27.9 | −3.8 | 0.227 | 0.5 |
| Tilt_half | 62.8 | 18.6 | 1982 | 5.6 | 28.9 | −0.5 | 0.237 | 4.7 |
| Tilt_double | 40.4 | −23.8 | 1835 | −2.2 | 28.3 | −2.4 | 0.227 | 0.5 |
| Roll_half | 78.8 | 48.7 | 2158 | 15.1 | 28.2 | −2.8 | 0.208 | −8.0 |
| Roll_double | 32.0 | −39.5 | 1749 | −6.8 | 29.9 | 2.9 | 0.188 | −16.7 |
| Twist_half | 53.0 | 0.1 | 1958 | 4.4 | 57.6 | 98.4 | 0.248 | 9.8 |
| Twist_double | 52.8 | −0.3 | 1928 | 2.8 | 13.3 | −54.1 | 0.249 | 10.3 |
| Shift-Slide_revsign^3^ | 52.9 | −0.1 | 1906 | 1.6 | 28.1 | −3.1 | 0.228 | 0.7 |
| Shift-Rise_revsign | 53.0 | 0.0 | 1968 | 4.9 | 28.4 | −2.0 | 0.224 | −0.8 |
| Shift-Tilt_revsign | 53.0 | 0.1 | 2019 | 7.7 | 28.9 | −0.3 | 0.215 | −4.9 |
| Shift-Roll_revsign | 53.0 | 0.0 | 1866 | −0.5 | 30.9 | 6.3 | 0.226 | 0.1 |
| Shift-Twist_revsign | 53.0 | 0.0 | 1934 | 3.1 | 26.5 | −8.5 | 0.216 | −4.7 |
| Slide-Rise_revsign | 53.1 | 0.2 | 2106 | 12.3 | 28.5 | −1.8 | 0.170 | −24.8 |
| Slide-Tilt_revsign | 52.9 | −0.1 | 1971 | 5.1 | 28.8 | −0.7 | 0.220 | −2.7 |
| Slide-Roll_revsign | 53.0 | 0.0 | 1889 | 0.7 | 31.4 | 8.2 | 0.262 | 15.7 |
| Slide-Twist_revsign | 53.0 | 0.1 | 1881 | 0.3 | 25.9 | −10.9 | 0.177 | −21.5 |
| Rise-Tilt_revsign | 53.1 | 0.1 | 1897 | 1.1 | 30.0 | 3.4 | 0.207 | −8.3 |
| Rise-Roll_revsign | 53.0 | 0.1 | 1684 | −10.2 | 29.7 | 2.3 | 0.216 | −4.3 |
| Rise-Twist_revsign | 53.0 | 0.0 | 1813 | −3.4 | 27.1 | −6.6 | −0.226 | −200.0 |
| Tilt-Roll_revsign | 53.1 | 0.1 | 1849 | −1.4 | 31.0 | 6.9 | 0.229 | 1.4 |
| Tilt-Twist_revsign | 52.9 | −0.1 | 1823 | −2.8 | 28.2 | −2.8 | 0.248 | 9.4 |
| Roll-Twist_revsign | 56.2 | 6.2 | 1910 | 1.8 | 25.4 | −12.5 | 0.234 | 3.4 |

^1^ Values for the original parameter set computed using the full simulation (see the ‘DNA_gau’ entry in Table 1).

^2^ Halving or doubling the variance of ‘shift’ parameter in the covariance matrix.

^3^ Reverse the sign of the shift-slide covariance in the covariance matrix.
