# Supplementary material for: Blind Predictions of DNA and RNA Tweezers Experiments with Force and Torque
Source: PLoS Comput Biol. 2014 Aug 7;10(8):e1003756. doi: 10.1371/journal.pcbi.1003756 (PMC4125081; doi:10.1371/journal.pcbi.1003756)
Supplement: Table S9 — Effect of individual parameters in covariance matrix for RNA. See captions of Table S9 for detailed explanations. (DOC) [file pcbi.1003756.s018.doc]

Table S9. Effect of individual parameters in covariance matrix for RNA.

|  | Bending persistence length (nm) | Change (%) | Stretch modulus (pN) | Change (%) | Torsional Persistence length (nm) | Change (%) | Link vs. force slope (rad/pN) | Change (%) |
| --- | --- | --- | --- | --- | --- | --- | --- | --- |
| Full^1^ | 66.3 |  | 965 |  | 51.8 |  | 0.162 |  |
| Original | 62.9 |  | 1063 | 0.0 | 52.8 |  | 0.174 |  |
| Shift_half^2^ | 63.0 | 0.1 | 1163 | 9.4 | 52.7 | −0.3 | 0.164 | −6.2 |
| Shift_double^2^ | 62.9 | 0.0 | 1115 | 4.9 | 58.8 | 11.3 | 0.161 | −7.6 |
| Slide_half | 62.9 | 0.0 | 1143 | 7.6 | 54.8 | 3.7 | 0.173 | −0.9 |
| Slide_double | 62.9 | 0.0 | 1106 | 4.0 | 52.8 | −0.1 | 0.176 | 0.7 |
| Rise_half | 62.9 | 0.0 | 1417 | 33.3 | 54.2 | 2.5 | 0.143 | −17.9 |
| Rise_double | 62.9 | 0.0 | 797 | −25.0 | 53.7 | 1.6 | 0.152 | −12.6 |
| Tilt_half | 73.7 | 17.1 | 1120 | 5.4 | 51.3 | −3.0 | 0.155 | −10.9 |
| Tilt_double | 48.7 | −22.6 | 1157 | 8.9 | 53.9 | 2.0 | 0.172 | −1.6 |
| Roll_half | 92.1 | 46.3 | 1422 | 33.9 | 55.9 | 5.8 | 0.237 | 35.9 |
| Roll_double | 38.5 | −38.7 | 854 | −19.6 | 49.3 | −6.6 | 0.047 | −73.2 |
| Twist_half | 64.0 | 1.7 | 1125 | 5.9 | 102.9 | 94.8 | 0.122 | −29.9 |
| Twist_double | 60.8 | −3.3 | 1109 | 4.4 | 28.0 | −47.1 | 0.316 | 81.1 |
| Shift-Slide_revsign^3^ | 63.0 | 0.1 | 1153 | 8.5 | 54.0 | 2.3 | 0.161 | −7.8 |
| Shift-Rise_revsign | 63.2 | 0.4 | 1132 | 6.5 | 54.2 | 2.7 | 0.174 | −0.1 |
| Shift-Tilt_revsign | 63.0 | 0.1 | 1137 | 7.0 | 52.6 | −0.4 | 0.151 | −13.4 |
| Shift-Roll_revsign | 62.9 | 0.0 | 1179 | 10.9 | 52.8 | 0.0 | 0.154 | −11.8 |
| Shift-Twist_revsign | 62.8 | −0.1 | 1142 | 7.5 | 54.7 | 3.6 | 0.151 | −13.3 |
| Slide-Rise_revsign | 62.9 | 0.1 | 1149 | 8.2 | 53.5 | 1.3 | 0.174 | −0.1 |
| Slide-Tilt_revsign | 62.9 | −0.1 | 1160 | 9.1 | 53.6 | 1.4 | 0.191 | 9.5 |
| Slide-Roll_revsign | 62.8 | −0.2 | 1149 | 8.1 | 55.5 | 5.0 | 0.166 | −4.5 |
| Slide-Twist_revsign | 62.9 | 0.0 | 1066 | 0.3 | 54.0 | 2.3 | 0.071 | −59.3 |
| Rise-Tilt_revsign | 62.9 | 0.0 | 1182 | 11.2 | 53.2 | 0.7 | 0.167 | −4.3 |
| Rise-Roll_revsign | 62.8 | −0.1 | 870 | −18.2 | 54.0 | 2.2 | 0.151 | −13.6 |
| Rise-Twist_revsign | 62.9 | 0.0 | 1081 | 1.7 | 54.4 | 3.0 | 0.100 | −42.8 |
| Tilt−Roll_revsign | 62.9 | −0.1 | 1106 | 4.1 | 51.1 | −3.3 | 0.179 | 2.6 |
| Tilt-Twist_revsign | 63.0 | 0.2 | 1167 | 9.8 | 55.0 | 4.1 | 0.167 | −4.0 |
| Roll-Twist_revsign | 68.0 | 8.0 | 1070 | 0.7 | 46.3 | −12.4 | 0.048 | −72.2 |

^1^ Values for the original parameter set computed using the full simulation (see the ‘RNA_gau’ entry in Table 1).

^2^ Halving or doubling the variance of ‘shift’ parameter in the covariance matrix.

^3^ Reverse the sign of the shift-slide covariance in the covariance matrix.
