# Supplementary material for: Blind Predictions of DNA and RNA Tweezers Experiments with Force and Torque
Source: PLoS Comput Biol. 2014 Aug 7;10(8):e1003756. doi: 10.1371/journal.pcbi.1003756 (PMC4125081; doi:10.1371/journal.pcbi.1003756)
Supplement: Table S10 — Standard deviations of parameters for random DNA, poly(A)/poly(T) and Z-DNA. 1 Z-DNA has a minimum repetitive unit of two base-pairs, therefore it has two distinct step parameter set (GC and CG). (DOC) [file pcbi.1003756.s019.doc]

Table S10. Standard deviations of parameters for random DNA, poly(A)/poly(T) and Z-DNA.

|  | Shift (Å) | Slide (Å) | Rise (Å) | Tilt (°) | Roll (°) | Twist (°) |
| --- | --- | --- | --- | --- | --- | --- |
| Random DNA, default | 0.57 | 0.86 | 0.23 | 3.56 | 5.17 | 6.24 |
| Random DNA, 2.8_all | 0.64 | 0.82 | 0.25 | 3.85 | 6.25 | 5.43 |
| Poly(A)/poly(T), default | 0.30 | 0.25 | 0.17 | 2.56 | 3.59 | 3.34 |
| Poly(A)/poly(T), 2.8_all | 0.47 | 0.49 | 0.21 | 3.11 | 6.73 | 5.45 |
| Z-DNA, GC^1^ | 0.27 | 0.26 | 0.19 | 2.25 | 2.70 | 3.21 |
| Z-DNA, CG^1^ | 0.20 | 0.18 | 0.21 | 2.22 | 3.29 | 2.97 |

^1^ Z-DNA has a minimum repetitive unit of two base-pairs, therefore it has two distinct step parameter set (GC and CG).
