# Supplementary material for: Blind Predictions of DNA and RNA Tweezers Experiments with Force and Torque
Source: PLoS Comput Biol. 2014 Aug 7;10(8):e1003756. doi: 10.1371/journal.pcbi.1003756 (PMC4125081; doi:10.1371/journal.pcbi.1003756)
Supplement: Table S11 — Acceptance rate of the Monte Carlo simulations. Link constrained simulations are performed at 7 pN stretching force. (DOC) [file pcbi.1003756.s020.doc]

Table S11. Acceptance rate of the Monte Carlo simulations.

|  | Force (pN) | Acceptance Rate (%) |
| --- | --- | --- |
| D N A | 1 | 54.9 |
|  | 10 | 18.9 |
|  | 20 | 12.6 |
|  | 40 | 8.1 |
| R N A | 1 | 63.5 |
|  | 10 | 24.9 |
|  | 20 | 16.6 |
|  | 40 | 10.6 |
|  | Target link (turns) | Acceptance Rate (%) |
| D N A | -8 | 19.1 |
|  | 0 | 19.1 |
|  | 8 | 18.9 |
| R N A | -8 | 26.8 |
|  | 0 | 26.7 |
|  | 8 | 24.2 |

Link constrained simulations are performed at 7 pN stretching force.
