# Supplementary material for: Blind Predictions of DNA and RNA Tweezers Experiments with Force and Torque
Source: PLoS Comput Biol. 2014 Aug 7;10(8):e1003756. doi: 10.1371/journal.pcbi.1003756 (PMC4125081; doi:10.1371/journal.pcbi.1003756)
Supplement: Table S12 — Values and sampling error of observables in HelixMC simulations at difference forces and link-constraints. The values in parenthesis are the corresponding sampling errors. (DOC) [file pcbi.1003756.s021.doc]

Table S12. Values and sampling error of observables in HelixMC simulations at difference forces and link-constraints.

|  | | Force extension simulations  Stretching force (pN) | | | Link-constrained simulations  Target link constraint (turn) | | |
| --- | --- | --- | --- | --- | --- | --- | --- |
|  |  | 0.04 | 4 | 40 | −8 | 0 | 8 |
| DNA | Extension (nm) | 330.0(0.752) | 928.6(0.031) | 992.7(0.004) | 942.5(0.009) | 946.5(0.009) | 949.9(0.010) |
|  | Link (rad) | 1845(0.038) | 1847(0.030) | 1869(0.012) | 1797(0.0003) | 1848(0.0003) | 1899(0.0003) |
| RNA | Extension (nm) | 317.4(0.702) | 781.1(0.027) | 845.4(0.004) | 789.7(0.009) | 796.4(0.009) | 802.7(0.009) |
|  | Link (rad) | 1710(0.028) | 1712(0.020) | 1729(0.008) | 1664(0.0003) | 1714(0.0003) | 1764(0.0003) |

The values in parenthesis are the corresponding sampling errors.
