# Supplementary material for: Blind Predictions of DNA and RNA Tweezers Experiments with Force and Torque
Source: PLoS Comput Biol. 2014 Aug 7;10(8):e1003756. doi: 10.1371/journal.pcbi.1003756 (PMC4125081; doi:10.1371/journal.pcbi.1003756)
Supplement: Table S13 — Covariance matrices for DNA and RNA default parameter sets. Data are derived from crystallographic models excluding proteins and with diffraction resolutions of 2.8 Å or better. Additional data sets, including sequence-dependent parameters, are available in the HelixMC package (http://github.com/fcchou/helixmc). (DOC) [file pcbi.1003756.s022.doc]

Table S13. Covariance matrices for DNA and RNA default parameter sets.

| D N A |  | Shift (Å) | Slide (Å) | Rise (Å) | Tilt (°) | Roll (°) | Twist (°) |
| --- | --- | --- | --- | --- | --- | --- | --- |
|  | Shift (Å) | 0.330 | 0.008 | −0.004 | 0.550 | 0.023 | −0.138 |
|  | Slide (Å) | 0.008 | 0.731 | 0.022 | −0.020 | −0.387 | 1.315 |
|  | Rise (Å) | −0.004 | 0.022 | 0.053 | 0.038 | −0.191 | 0.538 |
|  | Tilt (°) | 0.550 | −0.020 | 0.038 | 12.691 | −0.123 | 0.544 |
|  | Roll (°) | 0.023 | −0.387 | −0.191 | −0.123 | 26.696 | −13.449 |
|  | Twist (°) | −0.138 | 1.315 | 0.538 | 0.544 | −13.449 | 38.957 |
| R N A |  | Shift (Å) | Slide (Å) | Rise (Å) | Tilt (°) | Roll (°) | Twist (°) |
|  | Shift (Å) | 0.323 | 0.007 | −0.001 | 0.586 | 0.054 | −0.018 |
|  | Slide (Å) | 0.007 | 0.153 | −0.015 | −0.002 | 0.162 | 0.671 |
|  | Rise (Å) | −0.001 | −0.015 | 0.041 | −0.020 | 0.149 | 0.092 |
|  | Tilt (°) | 0.586 | −0.002 | −0.020 | 8.178 | 0.066 | −0.185 |
|  | Roll (°) | 0.054 | 0.162 | 0.149 | 0.066 | 18.723 | −2.327 |
|  | Twist (°) | −0.018 | 0.671 | 0.092 | −0.185 | −2.327 | 18.017 |

Data are derived from crystallographic models excluding proteins and with diffraction resolutions of 2.8 Å or better. Additional data sets, including sequence-dependent parameters, are available in the HelixMC package (<http://github.com/fcchou/helixmc>).
