# Supplementary material for: Blind Predictions of DNA and RNA Tweezers Experiments with Force and Torque
Source: PLoS Comput Biol. 2014 Aug 7;10(8):e1003756. doi: 10.1371/journal.pcbi.1003756 (PMC4125081; doi:10.1371/journal.pcbi.1003756)
Supplement: Table S14 — Comparison of simulations with default random sequence and a single random sequence. (DOC) [file pcbi.1003756.s023.doc]

Table S14. Comparison of simulations with default random sequence and a single random sequence.

| Simulations | *A*: bending persistence  (nm) | *S*: stretch modulus S(pN) | *C*: torsional persistence length(nm) | Slope of link vs. force (rad/pN) | Slope of extension vs. link (nm/turn) | *g*: link-extension coupling 1 (pN·nm) | *g*: link-extension coupling 2 (pN·nm) |
| --- | --- | --- | --- | --- | --- | --- | --- |
| RNA, default random sequence | 66.3(0.9) | 979.0(40.5) | 53.0(0.2) | 0.161(0.001) | 0.797(0.011) | −116.5(5.2) | −124.2(5.4) |
| RNA, single random sequence | 70.4(0.9) | 1228.8(51.6) | 55.6(0.2) | 0.139(0.001) | 0.740(0.014) | -132.7(6.1) | -144.7(6.7) |
