# Supplementary material for: Blind Predictions of DNA and RNA Tweezers Experiments with Force and Torque
Source: PLoS Comput Biol. 2014 Aug 7;10(8):e1003756. doi: 10.1371/journal.pcbi.1003756 (PMC4125081; doi:10.1371/journal.pcbi.1003756)
Supplement: Table S15 — Example computational time of HelixMC on a Linux desktop. Computer specification: Intel Core i7-3770 CPU @ 3.40 GHz, 24 GB RAM. Operation system: Linux Mint 13 Maya. Computation is performed using Enthought Python Distribution 7.3-1 academic edition. Each simulation uses a single thread and ∼700 MB of memory. All simulations are performed with 7 pN stretching force. (DOC) [file pcbi.1003756.s024.doc]

Table S15. Example computational time of HelixMC on a Linux desktop.

|  | Run1 | Run2 | Run3 | Run4 | Run5 | Run6 | Run7 |
| --- | --- | --- | --- | --- | --- | --- | --- |
| Number of base-pairs | 500 | 1,000 | 500 | 500 | 500 | 3,000 | 3,000 |
| Number of MC cycles | 1,000 | 1,000 | 2,000 | 1,000 | 1,000 | 1,000 | 1,000 |
| Link computation method | Fuller | Fuller | Fuller | Exact | Fuller | Fuller | Exact |
| Link constraint | No | No | No | No | Yes | No | No |
| Total time (s) | 84.8 | 192.35 | 161.3 | 104.8 | 152.0 | 717.0 | 1404.6 |

Computer specification: Intel Core i7-3770 CPU @ 3.40GHz, 24 GB RAM. Operation system: Linux Mint 13 Maya. Computation is performed using Enthought Python Distribution 7.3-1 academic edition. Each simulation uses a single thread and ~700 MB of memory. All simulations are performed with 7 pN stretching force.
