# Supplementary material for: Blind Predictions of DNA and RNA Tweezers Experiments with Force and Torque
Source: PLoS Comput Biol. 2014 Aug 7;10(8):e1003756. doi: 10.1371/journal.pcbi.1003756 (PMC4125081; doi:10.1371/journal.pcbi.1003756)
Supplement: Text S1 — Supplementary Methods. (DOC) [file pcbi.1003756.s025.doc]

# Supplementary Methods

## Clustering of DNA base-pair step parameters

During the curation of base-pair step parameters of DNA, we noticed that there are two major clusters in the distribution, most clearly visible from the slide distribution (Fig. S7). These clusters correspond to A-DNA and B-DNA in the crystallographic database, where B-DNA near-zero average slide and A-DNA has a negative average slide . To separate out the B-DNA conformations that are being studied in experiments, we applied the k-means clustering algorithm to the data. Here each data point can be represented as a six-dimensional vector. Since the six base-pair step parameters are measured in different units (Å for distances and radian for angles), we first rescaled the data such that all parameters have mean values of zero and standard deviation of one. To further improve the clustering performance, we increased the standard deviation of slide to 1.2, since slide is the most discriminating parameter for separating B-DNA and A-DNA. With k = 3, we could cleanly separate the two different DNA conformations, with one cluster representing the A-DNA and the other two clusters representing B-DNA (Fig. S7).

## Symmetry of the base-pair step parameter set

As discussed in the Methods section of the main text, we classify the base-pair step parameters according to the sequence of the first strand into 16 different categories, such as 5´-AT-3´/5´-AT-3´ (we will write AT/AT for simplification from now on), CA/TG, etc. However, the base-pair step parameters for a base-pair step can also be evaluated using the other strand. For example, CA/TG can also be evaluated as TG/CA, and AT/AT is self-symmetric. As pointed out by El Hassan and Calladine , computing the base-pair step parameter in such a reversed way does not change the magnitude of the parameter, but leads to a sign change of the shift and tilt. Therefore for each base-pair step parameter set from the database, one can reverse the sign of the shift and tilt, and assign it to the 16 sequence categories according to the sequence of the other strand. This symmetrization process leads to 10 independent sequence categories (AA/TT, GG/CC, AG/CT, AC/GT, GA/TC, CA/TG, AT/AT, GC/GC, TA/TA, CG/CG). Here each of the first six sequences corresponds to two sequences in the 16-category representation. For example, CA/TG and TG/CA are both represented by CA/TG. The last four sequences are self-symmetric, so each one of them corresponds to one sequence in the 16-category representation. This symmetrization process has the following consequences. First, non-self-symmetric sequence pairs (e.g. CA/TG and TG/CA) are represented by the same set of data, so their average values and covariance matrix have the same magnitude. The average shift and slide have different signs. Also all the cross terms in the covariance matrix involving either shift or tilt (shift-rise, tilt-slide, etc., except for tilt-shift that involves both) have different signs. Second, self-symmetric sequences (e.g. AT/AT and CG/CG) must have zero average shift and tilt, and zero covariance for parameter pairs that involves either shift or tilt (but not both, i.e. shift-tilt covariance is not necessarily zero). Third, if we represent the random-sequence DNA using a single multivariate Gaussian using all the symmetrized data, this distribution is self-symmetric and has the same behavior as the self-symmetric sequence dataset.

By default we did not apply the above symmetrization procedure in HelixMC to enable estimation of systematic errors due to any asymmetries in strand sequence dependent database. The symmetrization can be turned on by adding a flag “-symmetrize” to the command line. To investigate the effect of the symmetrization process on the computed mechanical properties, we repeated some of the simulations with the symmetrization enabled (Table S2). For random-sequence simulations, the symmetrization does not introduce any significant changes in the predicted mechanical properties. For simulations of poly(A)/poly(T), the symmetrization leads to changes of the bending and torsional persistence length in the range of 5-12 nm, and 100-200 pN changes of the stretching modulus. These differences give a lower bound of the systematic error in our parameter set.

## Details of the HelixMC calculations

All calculations in this work were performed on the TACC Ranger and Stampede cluster computers of the XSEDE resources. The temperature was set to 298.15 K (25 °C). For the force-extension simulations, we simulated the 3 kbp system at different stretching forces in parallel. We performed simulations for 16 different forces, distributed evenly in log scale in the range of 0.04 pN to 40 pN. For simulations with applied forces lower than or equal to 4 pN, the exact writhe formula was used to compute the link of the system. In other cases we used the faster Fuller method to compute the link. The validity of the Fuller method at forces > 4 pN has been demonstrated in the main text. For simulations with exact link computation, we obtained 40,000 samples in total for each force simulated (each sample was extracted at the end of each MC cycle; see Methods section of main text). For simulations with Fuller link computation, which is faster, we obtained 240,000 samples for each force. For each force, the sampling was parallelized by running 20 independent HelixMC jobs, each giving 1/20 of the total data.

For link-constrained simulations, we first performed a 500-cycle pre-run at 7 pN stretching force and no link constraint, to obtain the average link for the helix at a relaxed state. This link value was then used as the zero-point for further link-constraint simulations. For each parameter set, we simulated the system at ten different target link-constraints, ranging from -8 turns to 10 turns with an interval of 2 turns. The stretching forces were all set to 7 pN. For each target link-constraint, 140,000 samples in total were obtained from the simulation, again by running 20 independent jobs.

## Example HelixMC command lines

Here we list command line examples for the HelixMC package. For a more detailed description on the installation and usage of HelixMC, please refer to the online HelixMC documentation (<http://fcchou.github.com/HelixMC/>).

1. Display help message.

helixmc-run --help

1. Use the DNA_default parameter set to simulate a 500 bp helix under 10 pN stretching force. Perform 500 full-helix MC update steps. Compute and record the link of the helix using Fuller’s approximate writhe formula.

helixmc-run -params DNA_default.npz -n_bp 500 -n_step 100 -force 10 -compute_fuller_link

1. Similar to example 2, but the sequence is specified to be poly(A). The link of helix is constrained to stay close to 310 rad, with a trap stiffness of 2,000 pN·Å·rad^-1^. The link of helix is computed using the exact formula instead. Also the sampling scheme is set to fragment picking instead of assuming multivariate Gaussian distributions.

helixmc-run -params DNA_default.npz -n_bp 500 -n_step 100 -force 10 -seq A -target_link 310 -torsional_stiffness 2000 -compute_exact_link –no_gaussian_sampling

1. Simulate 500 bp helix for 100 steps using the RNA_gau single Gaussian parameter set. The stretching force is zero.

helixmc-run -gaussian_params RNA_gau.npy -n_bp 500 -n_step 100

## Fitting functions used in this work

Here we summarized the fitting functions used in analyzing the simulation data in this work. All the functions listed below are implemented in the HelixMC package, and can be combined with the fitting utility in the SciPy python module to fit a given dataset.

1. Inextensive worm-like chain model by Bouchiat et al. .

$$\begin{matrix} F=\frac{k_{B}T}{A}\left[\frac{1}{4(1-z/L)^{2}}-\frac{1}{4}+\frac{z}{L}+\sum_{i=2}^{i\leq7} \alpha_{i}\left(\frac{z}{L}\right)^{i}\right] \\ \alpha_{i}=\text{[-0.5164228, -2.737418, 16.07497, -38.87607, 39.49944, -14.17718]} \\ \text{for }i\in[2,7]\text{ } \end{matrix}$$

Here *F* is the stretching force, *A* is the bending persistence length, *L* is the helix contour length, *k_B_* is the Boltzmann constant, *T* is the temperature, *z* is the helix extension, and *α_i_* are a set of constants defined above.

1. Extensive worm-like chain model by Bouchiat et al. .

$$\begin{matrix} F=\frac{k_{B}T}{A}\left(\frac{1}{4(1-l)^{2}}-\frac{1}{4}+l+\sum_{i=2}^{i\leq7} \alpha_{i}l^{i}\right) \\ l=z/L-F/S \end{matrix}$$

Note that this is an implicit function of *F.*

1. Extensive worm-link chain model by Odijk .

$$z=L\left(1-\frac{1}{2}\sqrt{\frac{k_{B}T}{FA}}+\frac{F}{S}\right)$$

1. Relation between torque and link in link-constrained experiments .

$$\tau=\frac{C_{eff}k_{B}T}{L}\left\langle\Delta Lk\right\rangle$$

Here *τ* is the applied torque, <*ΔLk*> is the average change of link, and *C_eff_* is the effective torsional persistence length. At the high-force limit, we have *C_eff_* equals *C*, the actual torsional persistence length. In this case we have

$$\tau=\frac{Ck_{B}T}{L}\left\langle\Delta Lk\right\rangle$$

This is the equation we used to obtain torsional persistence length from the slope of torque vs. constrained link in Table S7.

1. Moroz-Nelson fitting function .

$\begin{matrix} C_{eff}=C\left(1-\frac{M}{4K_{0}}+\frac{M^{2}-2M}{16K_{0}^{2}}-\frac{4M^{3}-16M^{2}+21M}{256K_{0}^{3}}\right) \\ \text{with }M=\frac{C}{A}, K_{0}=\sqrt{\frac{AF}{k_{B}T}} \end{matrix}$

Here *C* is the torsional persistence length and *C_eff_* is the effective torsional persistence length. We used here the expansion up to the 3^rd^ order.

Eq. is derived from the original Moroz-Nelson equation using the Taylor expansion . Below we repeat the derivation for the reader’s reference.

The original Moroz-Nelson equation (Eq. 61 in ref. ) is

$$\begin{matrix} \omega_{o}\sigma=\frac{\tau}{k_{B}T}\left[\frac{1}{C}+\frac{1}{4AK}+\frac{1}{8AK^{2}}+\frac{21}{256AK^{3}}+O(K^{4})\right] \\ K=\sqrt{\frac{AF}{k_{B}T}-\frac{\tau^{2}}{4(k_{B}T)^{2}}} \end{matrix}$$

Here *ω*_0_ is the helical density of relaxed molecule, *σ* is the helix overtwist, and *τ* is the applied torque. The changes of average helix link can be related to the helix overtwist using

$$\left\langle\Delta Lk\right\rangle=\omega_{0}\sigma L$$

Here *L* is the helix contour length. Combining Eq. and , we have

$$\left\langle\Delta Lk\right\rangle=\frac{\tau L}{k_{B}T}\left[\frac{1}{C}+\frac{1}{4AK}+\frac{1}{8AK^{2}}+\frac{21}{256AK^{3}}+O(K^{4})\right]$$

This original Moroz-Nelson equation describes the relation between torque and changes of link in link-constrained experiment. At high force and low torque, we have

$$K=\sqrt{\frac{AF}{k_{B}T}-\frac{\tau^{2}}{4(k_{B}T)^{2}}}\approx\sqrt{\frac{AF}{k_{B}T}}=K_{0}$$

Therefore,

$$\left\langle\Delta Lk\right\rangle\approx\frac{\tau L}{k_{B}T}\left[\frac{1}{C}+\frac{1}{4AK_{0}}+\frac{1}{8AK_{0}^{2}}+\frac{21}{256AK_{0}^{3}}\right]$$

Which leads to a linear relationship between torque and link as observed in both experiments and simulations.

Combining Eq. and , we have

$$\begin{matrix} C_{eff}=\left[\frac{1}{C}+\frac{1}{4AK_{0}}+\frac{1}{8AK_{0}^{2}}+\frac{21}{256AK_{0}^{3}}\right]^{-1} \\ =C\left[1+\frac{C}{4AK_{0}}+\frac{C}{8AK_{0}^{2}}+\frac{21C}{256AK_{0}^{3}}\right]^{-1} \\ =C\left[1+\frac{M}{4K_{0}}+\frac{M}{8K_{0}^{2}}+\frac{21M}{256K_{0}^{3}}\right]^{-1} \end{matrix}$$

Here *M = C / A*. At high force limit, as *F* → *∞*, 1 / *K*_0_ → 0. By performing Taylor expansion of Eq. at
1 / *K*_0_ = 0, and only keep terms up to *K*_0_^-3^, we obtain Eq. .

## Fast evaluation for mechanical properties

To test the effect of varying each individual parameters in the covariance matrix, we applied a rapid method to evaluate the mechanical properties corresponds to a modified parameter dataset, without performing full simulations on a helix of 3 kbp. Here we evaluated the bending persistence length using Olson’s matrix averaging approach , which gives an accurate estimation for bending persistence length in less than 10 seconds. The stretch modulus is evaluated using the slope of the force-extension curve at high forces on ultra-short helices. At this condition the helix bending is negligible and the force-extension curve is linear. Here we simulated helices of 20 base-pairs at 100 pN and 120 pN stretching force, and calculated the slope using these two data points. The slope is related to the stretch modulus by the relation $z=\frac{L}{S}F$, which is essentially the main text Eq. (32) with zero link-extension coupling. To quickly evaluate the torsional persistence length and link-extension coupling, we simulated helices with 500 base-pairs under constant stretch forces, at 2 pN and 16 pN. We estimated the torsional persistence lengths using the fact that the effective torsional persistence lengths from the 16 pN simulations are a good estimate for the true torsional persistence length. The link-extension coupling was evaluated similarly by fitting a straight line to the link vs. force plot, but here we only had two data points so the slope was directly calculated without fitting. To ensure this fast evaluation scheme gave reasonable estimations, we compared the values computed using the fast method and using the original full simulation for the single Gaussian dataset (DNA_gau/RNA_gau), and the obtained values agreeing within 6 %.

1. Olson WK, Gorin AA, Lu X-J, Hock LM, Zhurkin VB (1998) DNA sequence-dependent deformability deduced from protein–DNA crystal complexes. Proceedings of the National Academy of Sciences 95: 11163-11168.

2. El Hassan MA, Calladine CR (1995) The Assessment of the Geometry of Dinucleotide Steps in Double-Helical DNA; a New Local Calculation Scheme. Journal of Molecular Biology 251: 648-664.

3. Bouchiat C, Wang MD, Allemand J, Strick T, Block SM, et al. (1999) Estimating the persistence length of a worm-like chain molecule from force-extension measurements. Biophysical Journal 76: 409-413.

4. Odijk T (1995) Stiff Chains and Filaments under Tension. Macromolecules 28: 7016-7018.

5. Moroz JD, Nelson P (1997) Torsional directed walks, entropic elasticity, and DNA twist stiffness. Proceedings of the National Academy of Sciences 94: 14418-14422.

6. Moroz JD, Nelson P (1998) Entropic Elasticity of Twist-Storing Polymers. Macromolecules 31: 6333-6347.

7. Gore J (2005) Single-molecule studies of DNA twist mechanics and gyrase mechanochemistry [Ph. D. Thesis]: University of California, Berkeley.

8. Olson WK, Colasanti AV, Czapla L, Zheng G (2008) Insights into the Sequence-Dependent Macromolecular Properties of DNA from Base-Pair Level Modeling. In: Voth GA, editor. Coarse-Graining of Condensed Phase and Biomolecular Systems: CRC Press. pp. 205-223.
