# Supplementary material for: Blind Predictions of DNA and RNA Tweezers Experiments with Force and Torque
Source: PLoS Comput Biol. 2014 Aug 7;10(8):e1003756. doi: 10.1371/journal.pcbi.1003756 (PMC4125081; doi:10.1371/journal.pcbi.1003756)
Supplement: Text S2 — Supplementary Results. (DOC) [file pcbi.1003756.s026.doc]

# Supplementary Results

## The origin of stretch modulus differences in dsRNA and dsDNA

To better understand why RNA helices consistently gave a lower stretch modulus than DNA helices in both experiments and simulation, we explored a geometric hypothesis that the “springy” axis curve (Fig. 2) renders RNA duplexes more pliable to force, analogous to a spring’s low stretch modulus compared to a straight wire. To test this hypothesis, we performed additional simulations with two artificial datasets by modifying the single-Gaussian datasets for DNA and RNA (Table S4). The first dataset, named “DNA_gau_graft”, has the same mean six-parameters as DNA, but with the covariance matrix coming from the RNA dataset; it simulates a molecule with the same equilibrium ‘wire-like’ shape as DNA helices but with the microscopic base-pair-level flexibility of RNA. Replacing the covariance matrix of DNA with that of RNA does not lower its stretch modulus and indeed slightly increases it. A second simulation used the mean six parameters of RNA and the covariance matrix of DNA (“RNA_gau_graft”); this simulation retained the softer (lower) stretch modulus of the RNA simulations. Both simulations support a model in which the equilibrium geometry, not the microscopic fluctuations of the six-parameter distribution, explains the softer stretch modulus of RNA compared to DNA. The above results confirms our hypothesis that the “springy” equilibrium axis curve indeed plays a critical role in the low stretch modulus of RNA. When the polymers are over-stretched, for DNA the extension mostly goes to the increase of rise, but for RNA the extension is absorbed by both changes in rise and roll, making RNA softer than DNA (Table S5). To further quantify the degree of “springiness” in the simulated conformations, we computed the sine of super-helical pitch angle *α* as follows:

$$\sin\alpha=\frac{L_{eff}}{L_{axis}}$$

Here *L_axis_* is the length of the axis curve (the red lines in Fig. 2). *L_eff_* is the effective helix contour length, defined as follows:

$$L_{eff}=(1+F/S)L$$

Here *F* is the applied stretching force, *S* is the stretch modulus, and *L* is the helix contour length. The effective contour length is the helix contour length plus the contribution of direct helix stretching upon external force, so it is equivalent to the helix extension when the helix has no bending. For cases in which the helix axis curve is completely straight, the length of the axis curve is equal to the effective contour length, and sin*α* is 1. On the other hand, a winding axis curve (typical in dsRNA) is longer than the effective contour length, leading to smaller sin*α* (between 0 and 1). Here we found that upon increasing stretching forces, the sin*α* of dsDNA stays mostly constant, while the sin*α* of dsRNA increases with higher stretching force (Table S5). This result further supports the axis curve of dsRNA is unwound upon stretching, leading to a smaller “springiness”.

## Checking the percentage of steric clashed conformations

To check the percentage of steric clashed conformations, we recorded the axis-curve coordinates of all frames in a 1,000-step simulation (using the -save_all_frames flag in the helixmc-run command line). For each frame, we computed all the pair-wise distances between the base-pair centers separated by more than 20 base-pairs (short-range distances are dictated by local helix geometry and provide no information for long-range clashes). If any of the pair-wise distances was smaller than 25 Å, the conformation is considered to be clashed. The distance cutoff is selected to be slightly larger than the diameter of the helix (~ 19 Å, see ref ) to account for the van der Waals interaction cutoff. The results (Table S6) show that at zero stretching force, a small portion of the conformations have steric clashes (5.6 % for dsDNA, 2.8 % for dsRNA). However with even a small stretching force (0.4 pN), for both dsDNA and dsRNA there is no steric clashed conformation in all the 1,000 frames simulated. This result suggests that steric clashes can be safely neglected for the simulations performed in this work, although it may be critical for expanding HelixMC to simulate more collapsed helix states like [plectoneme](https://www.google.com/search?rlz=1C1CHFX_enUS558US558&es_sm=93&qscrl=1&biw=1280&bih=632&q=plectoneme&spell=1&sa=X&ei=NE1YU6vVE4yTyASD0ILIDA&ved=0CCkQvwUoAA)s.

## Relation of two methods for link-extension measurements

To convert the observed slopes to the more physically-relevant link-extension coupling constant, previous work by Gore and colleagues used a simple linear unbendable rod model. Here we will repeat the derivation before moving on to further discussion.

First, we treat the nucleic acid helix as an isotropic elastic rod that does not bend. Under an external stretching force *F*, the energy of the rod can be expanded as:

$$E=\frac{1}{2}k_{B}T\frac{C}{L}\theta^{2}+\frac{1}{2}\frac{S}{L}z^{2}+g\theta\frac{z}{L}-zF$$

Here, *C* is the torsional persistence length, *S* is the stretch modulus, *g* is the link-extension coupling constant, *F* is the external stretching force, *L* is the contour length, *θ* is the link of the rod, *z* is the rod extension, *k_B_* is the Boltzmann constant, and *T* is the temperature. To compute the slope observed in variable-force experiment, we set *F* to be a constant in Eq. , and minimize with respect to *z* and *θ*. We then obtain

$$\begin{matrix} \left(\frac{\partial E}{\partial z}\right)_{F,\theta}=\frac{S}{L}z+\frac{g}{L}\theta-F=0 \\ \left(\frac{\partial E}{\partial\theta}\right)_{F,\mathrm{z}}=k_{B}T\frac{C}{L}\theta+\frac{g}{L}z=0 \end{matrix}$$

Solving the equations, we obtain:

$$\theta=\frac{L}{g-k_{B}TCS/g}F$$

Here *θ* corresponds to the observed link changes in experiments, therefore Eq. predicts a linear relationship between the link changes vs. applied forces.

Similarly, for link-constrained experiment, we set both *F* and *θ* to be constants and minimize *z*. The final result is a linear relationship between the applied link constraint and the helix extension:

$$z=-\frac{g}{S}\theta+\frac{FL}{S}$$

We can derive the following linear force-extension relation from Eq. as well.

$$z=\frac{L}{S-\frac{g^{2}}{k_{B}TC}}F$$

Since the DNA/RNA helices bend significantly in the low-to-medium force regime (F < 1 pN), this unbendable rod model is only applicable at high-force limit. Indeed, by examining the force extension curve in our simulations, we found the linear relationship in Eq. holds only when stretching force > 15 pN (Fig. S3). However, in both experiments and our simulations, the linear relationship between changes in linking number and applied force holds at force as low as 1 pN, which is well below the high-force limit of the force-extension curve (Fig. 5A-B) . The link constraint vs. helix extension simulations, performed at 7 pN stretching force, gave a near-perfect linear relationship as well (Fig. 5C-D); experiments that showed the same linear relationship were also performed at similar stretching forces . Moreover, if we computed the link-extension coupling constant *g* in both setups using Eq. and , the obtained values agreed well with each other in both experiments and simulations (Table 2, Fig. S4 and ref. ). This simple unbendable rod model, which fails to predict the force-extension curve behavior except at high-force limit (> 15 pN), successfully predicts the linear relation observed in link change vs. applied force experiments and link constraint vs. helix extension experiments over a wide force range, and correctly captures the relationship between slopes observed in these two different experiments. To our knowledge, none of the existing analytical models can correctly explain how non-linearities introduced by bending cancel to give the observed linear relationships in both types of link-extension experiments.

## Effect of each parameter in the covariance matrix of the Gaussian

Here we will apply HelixMC simulations to understand the effect of each parameter in the multivariate Gaussian distribution on the mechanical properties of DNA/RNA. We modified the single-Gaussian parameter set by doubling, halving or reversing the sign of each of the 21 independent parameters in the covariance matrix, then performed fast calculations to obtain the corresponding bending persistence length, stretch modulus, torsional persistence length and link-extension coupling of each artificial dataset. The results are shown in Table S8-S9. From the table, we found that the bending persistence length is predominantly affected by the variance of tilt and roll, as might be expected: tilt and roll are directly related to helix bending. The dominant factor controlling torsional persistence length is the variance of twist, which is quite intuitive as well. The stretch modulus for both DNA and dsRNA is unsurprisingly dependent on the variance of rise. The stretch modulus of dsRNA also depends on variance of roll, in agreement with our previous observation on how the “springiness” of dsRNA affects its stretch modulus. The link-extension coupling of DNA is predominantly affected by the twist-rise covariance, consistent with arguments of a previous work . However, it appears that the link-extension coupling of dsRNA depends on multiple factors, in which the most significant ones include the covariance of twist-rise, twist-roll, twist-slide, and the variance of twist and roll. We argue that because dsRNA is more “springy” than DNA, the effect of stretching is shared by changes in rise, slide and roll, therefore the link-extension coupling is strongly affected by the twist-rise, twist-roll and twist-slide covariance. However, we do not have an intuitive explanation for why the variance of twist and roll also significantly affects the link-extension coupling for dsRNA but not for DNA.

The above results can also be used to explain the apparent higher stiffness of poly(A)/poly(T) and Z-DNA found in our simulations. For poly(A)/poly(T) with the default parameter set, the variances of rise, tilt, roll and twist are all smaller than random DNA (Table S10). From the conclusions above, we expect poly(A)/poly(T) will have larger bending persistence (smaller tilt and roll variance), torsional persistence (smaller twist variance) and stretch modulus (smaller rise variance), which is what we observed from the full simulations. On the other hand, poly(A)/poly(T) with the 2.8_all parameter set has a similar rise and twist variance, smaller tilt variance and larger roll variance compared to random DNA, and so the calculated mechanical properties are not significantly different. For Z-DNA, the minimum repetitive unit has two base-pair steps. For both base-pair steps (GC step and CG step, Table S10), the variances of tilt, roll and twist are smaller than random DNA, therefore Z-DNA has large bending and torsional persistence length (Table S10). The link-extension coupling, as discussed above, depends on multiple factors in the covariance matrix. It is therefore difficult to derive a simple reason for why Z-DNA is predicted to give a near-zero link-extension coupling.

## Effect of multi-base-pair parameter set on predicted mechanical properties

To understand how correlations between non-nearest neighbor base-pairs may affect the predicted mechanical properties of dsDNA and dsRNA, we performed additional simulations using multi-base-pair parameter sets. These parameter sets are curated using crystal structures with resolution ≤ 2.8 Å, including protein-binding models. Unlike the “2.8_all” datasets, sequence dependence is neglected during the curation, since an n-base-pair parameter set has 4^n^ possible sequence combinations and it is impossible to account for such sequence-dependence when n is large. For each parameter set, we extracted all n-base-pair fragments (where n ranges from 2 to 7), computed the six step-parameters between the first and the last base-pairs in the fragments, and used these parameters in HelixMC sampling. As a control, for each parameter set where n ≥ 3, we created a control parameter set, where we randomly selected n parameters from the two-base-pair parameter set, combined them into a helix to obtain the step-parameter between the first and the last base-pairs, and repeated this process 100,000 times to generate all parameters in the parameter set. The control parameter set represents the case where there is no correlation between each parameter in the two-base-pair parameter set. The mechanical properties for each parameter set are computed by the method described in “Fast evaluation for mechanical properties” section of the Supplementary Methods.

From simulations using multi-base-pair parameter sets, we found that the correlation between base-pairs may have significant effects on the predicted mechanical properties (Fig. S5-S6). On the other hand, the predicted values from the control dataset are independent of the number of base-pairs in the parameter set, as expected. Here the torsional persistence length for both dsDNA and dsRNA increases as the number of base-pairs in the parameter set increases, leading to values closer to the experimental measurements (109 nm and 100 nm for dsDNA and dsRNA respectively). However, the stretch modulus decreases for dsDNA and increases for dsRNA with increasing number of correlated base-pairs, in contradiction to the experimental data. For bending persistence length and link-extension coupling, changing the number of base-pairs in the curated dataset also has a significant effect on the predicted values.

1. Bloomfield VA, Crothers DM, Tinoco I, Jr. (2000) Nucleic Acids: Structures, Properties, and Functions: University Science Books.

2. Gore J, Bryant Z, Nöllmann M, Le MU, Cozzarelli NR, et al. (2006) DNA overwinds when stretched. Nature 442: 836-839.

3. Lionnet T, Joubaud S, Lavery R, Bensimon D, Croquette V (2006) Wringing Out DNA. Physical Review Letters 96.

4. Lionnet T, Lankaš F (2007) Sequence-Dependent Twist-Stretch Coupling in DNA. Biophysical Journal 92: L30-L32.
